# Supplementary material for: Genome assemblies of Vigna reflexo-pilosa (créole bean) and its progenitors, Vigna hirtella and Vigna trinervia, revealed homoeolog expression bias and expression-level dominance in the allotetraploid
Source: Gigascience. 2023 Jul 20;12:giad050. doi: 10.1093/gigascience/giad050 (PMC10357499; doi:10.1093/gigascience/giad050)
Supplement: giad050_GIGA-D-23-00032_Original_Submission [file giad050_giga-d-23-00032_original_submission.pdf]

GigaScience

# Genome assemblies of Vigna reflexo-pilosa (créole bean) and its progenitors, Vigna hirtella and Vigna trinervia, revealed homoeolog expression bias and expression level dominance in the allotetraploid

--Manuscript Draft--

| | |
|---|---|
| **Manuscript Number:** | GIGA-D-23-00032 |
| **Full Title:** | Genome assemblies of Vigna reflexo-pilosa (créole bean) and its progenitors, Vigna hirtella and Vigna trinervia, revealed homoeolog expression bias and expression level dominance in the allotetraploid |
| **Article Type:** | Data Note |
| **Funding Information:** | National Science and Technology Development Agency (1000221) — Dr. Wirulda Pootakham |
| **Abstract:** | Vigna reflexo-pilosa (créole bean) is a wild legume belonging to the subgenus Ceratoropis and is widely distributed in Asia. Créole bean is the only tetraploid species in the genus Vigna, and it has been shown to derive from the hybridization of Vigna hirtella and Vigna trinervia. In this study, we combined the long-read PacBio technology with the chromatin contact mapping (Hi-C) technique to obtain a chromosome-level assembly of V. reflexo-pilosa. The final assembly contained 998,724,903 bases with an N50 length of 42,545,650 bases. Our gene prediction recovered 97.5% of the highly conserved orthologs based on the BUSCO analysis. To investigate homoeolog expression bias and expression level dominance in the tetraploid, we also sequenced and assembled the genomes of its progenitors. Overall, the majority of the homoeolog pairs (72.9%) displayed no expression bias, and among those that exhibited biased expression, 16.3% showed unbalanced homoeolog expression bias toward the V. trinervia subgenome. Moreover, 41.2% and 36.2% of the expressed gene pairs exhibited transgressive expression and expression level dominance, respectively. Interestingly, the genome-wide expression level dominance in the tetraploid was biased toward the V. trinervia subgenome. The analysis of methylation patterns also revealed that the average methylation levels in coding regions were higher in the V. hirtella subgenome than those in the V. trinervia subgenome. The genomic/transcriptomic resources for these three species are useful not only for the development of elite cultivars in Vigna breeding programs but also to researchers studying comparative genomics and investigating genomic/epigenomic changes following polyploid events. |
| **Corresponding Author:** | Wirulda Pootakham<br>National Science and Technology Development Agency<br>Khlong Luang, Pathum Thani THAILAND |
| **Corresponding Author Secondary Information:** | |
| **Corresponding Author's Institution:** | National Science and Technology Development Agency |
| **Corresponding Author's Secondary Institution:** | |
| **First Author:** | Wirulda Pootakham |
| **First Author Secondary Information:** | |
| **Order of Authors:** | Wirulda Pootakham |
| | Chutima Sonthirod |
| | Chaiwat Naktang |
| | Chutintorn Yundaeng |
| | Thippawan Yoocha |
| | Wasitthee Kongkachana |
| | |

Powered by Editorial Manager® and ProduXion Manager® from Aries Systems Corporation

| | |
|---|---|
| | Duangjai Sangsrakru |
| | Prakit Somta |
| | Sithichoke Tangphatsornruang |
| **Order of Authors Secondary Information:** | |
| **Additional Information:** | |
| **Question** | **Response** |
| Are you submitting this manuscript to a special series or article collection? | No |
| **Experimental design and statistics**<br><br>Full details of the experimental design and statistical methods used should be given in the Methods section, as detailed in our Minimum Standards Reporting Checklist. Information essential to interpreting the data presented should be made available in the figure legends.<br><br>Have you included all the information requested in your manuscript? | Yes |
| **Resources**<br><br>A description of all resources used, including antibodies, cell lines, animals and software tools, with enough information to allow them to be uniquely identified, should be included in the Methods section. Authors are strongly encouraged to cite Research Resource Identifiers (RRIDs) for antibodies, model organisms and tools, where possible.<br><br>Have you included the information requested as detailed in our Minimum Standards Reporting Checklist? | Yes |
| **Availability of data and materials**<br><br>All datasets and code on which the conclusions of the paper rely must be either included in your submission or deposited in publicly available repositories (where available and ethically | Yes |

| | |
|---|---|
| appropriate), referencing such data using a unique identifier in the references and in the “Availability of Data and Materials” section of your manuscript.<br><br>Have you have met the above requirement as detailed in our Minimum Standards Reporting Checklist? | |

Powered by Editorial Manager® and ProduXion Manager® from Aries Systems Corporation

Manuscript

Click here to access/download;Manuscript;2023-02-03 Vigna reflexo genome assembly.docx

# Genome assemblies of *Vigna reflexo-pilosa* (créole bean) and its progenitors, *Vigna hirtella* and *Vigna trinervia*, revealed homoeolog expression bias and expression level dominance in the allotetraploid

Wirulda Pootakham[1,*], Chutima Sonthirod[1,†], Chaiwat Naktang[1,†], Chutintorn Yundaeng[1], Thippawan Yoocha[1], Wasitthee Kongkachana[1], Duangjai Sangsrakru[1], Prakit Somta[2], Sithichoke Tangphatsornruang[1]

[1]National Omics Center, National Science and Technology Development Agency (NSTDA), Pathum Thani, Thailand.

[2]Department of Agronomy, Faculty of Agriculture at Kamphaeng Saen, Kasetsart University, Nakhon Pathom, Thailand.

[†]Equal contribution

*Corresponding authors

Wirulda Pootakham (wirulda@alumni.stanford.edu)

**Running title:** Créole bean reference genome assembly

**Abstract**

*Vigna reflexo-pilosa* (créole bean) is a wild legume belonging to the subgenus *Ceratoropis* and is widely distributed in Asia. Créole bean is the only tetraploid species in the genus *Vigna*, and it has been shown to derive from the hybridization of *Vigna hirtella* and *Vigna trinervia.* In this study, we combined the long-read PacBio technology with the chromatin contact mapping (Hi-C) technique to obtain a chromosome-level assembly of *V. reflexo-pilosa*. The final assembly contained 998,724,903 bases with an N50 length of 42,545,650 bases. Our gene prediction recovered 97.5% of the highly conserved orthologs based on the BUSCO analysis. To investigate homoeolog expression bias and expression level dominance in the tetraploid, we also sequenced and assembled the genomes of its progenitors. Overall, the majority of the homoeolog pairs (72.9%) displayed no expression bias, and among those that exhibited biased expression, 16.3% showed unbalanced homoeolog expression bias toward the *V. trinervia* subgenome. Moreover, 41.2% and 36.2% of the expressed gene pairs exhibited transgressive expression and expression level dominance, respectively. Interestingly, the genome-wide expression level dominance in the tetraploid was biased toward the *V. trinervia* subgenome. The analysis of methylation patterns also revealed that the average methylation levels in coding regions were higher in the *V. hirtella* subgenome than those in the *V. trinervia* subgenome. The genomic/transcriptomic resources for these three species are useful not only for the development of elite cultivars in *Vigna* breeding programs but also to researchers studying comparative genomics and investigating genomic/epigenomic changes following polyploid events.

**Keywords:** *Vigna reflexo-pilosa*, chromosome-scale genome assembly, *Vigna hirtella, Vigna trinervia,* Hi-C, polyploidization, homoeolog expression bias, expression level dominance, bisulfite sequencing, methylation

## Data Description

### Context

*Vigna reflexo-pilosa* (*Vigna glabrescent*; créole bean) is one of the seven domesticated Asian *Vigna* species (subgenus *Ceratotropis*) (Maréchal, 1978;Tomooka et al., 2002) widely distributed across the Pacific islands, northern Australia and Papua New Guinea as well as in East, South and Southeast Asia (Tateishi, 1985;Tateishi and Ohashi, 1990). All Asian *Vigna* species have a diploid chromosome composition of $2n = 2x = 22$ whereas *V. reflexo-pilosa* is the only tetraploid species with a haploid chromosome number of 22 ($2n = 4x = 44$) (Swindell et al., 1973;Tomooka et al., 2002;Norihiko et al., 2010). *V. reflexo-pilosa* shows the formation of 22 bivalents during meiosis and is considered to be an amphidiploid (Egawa et al., 1990). A phylogenetic tree and principal coordinate analysis clearly demonstrated that *Vigna hirtella* and *Vigna trinervia* were the two genome donor parents (Chankaew et al., 2014). Interspecific hybridization (Egawa et al., 1996b) and analyses of plastid DNA sequences also suggested that *V. trinervia* is the maternal genome donor of *V. reflexo-pilosa* (Tun and Yamaguchi, 2007;Javadi et al., 2011). Créole bean is considered a novel crop for the future. In addition, wild accessions exhibit resistance to a number of insect pests and diseases such as bean fly, cucumber mosaic virus and powdery mildew and serve as a principal gene pool for improving the quality of cultivated varieties (Egawa et al., 1996a). As *V. reflexo-pilosa* can partially cross-pollinate with other *Vigna* species such as mungbean, it has become a potential genetic source for breeding *Vigna* species (Chankaew et al., 2014).

Several economic crop species are allopolyploids, including cotton (*Gossypium hirsutum*), tobacco (*Nicotiana tabacum*) and wheat (*Triticum aestivum*). Allopolyploids usually exhibit great vigor and adaptation to various biotic and abiotic stresses (Chen, 2007). The analyses of allopolyploid genomes demonstrated that one of the parental species' genomes (referred to as subgenomes) often had greater gene retention (Thomas et al., 2006), higher gene expression (Edger et al., 2019) and lower DNA methylation (Woodhouse et al., 2014). Furthermore, gene expression patterns of homoeologous genes (orthologous genes encoded on different parental

subgenomes) may change after the hybridization and polyploidization events, and these changes may yield phenotypic differences between the allopolyploid and their donor species (Doyle et al., 2008;Jackson and Chen, 2010). Homoeolog expression bias, where two homoeologs are expressed unequally, has the potential to greatly impact the phenotypic variation within a species. There have been examples of dominant subgenome contributing more to trait heritability than the non-dominant subgenome (Renny-Byfield et al., 2017) and controlling biological pathways related to agronomic traits (Edger et al., 2019).

In this study, we employed a combination of PacBio long-read sequencing technology with the *in vivo* fixation of chromosomes (Hi-C) technique to generate the first chromosome-scale assembly of *V. reflexo-pilosa*. We also obtained high-quality assemblies of the progenitor genomes, *V. hirtella* and *V. trinervia* using the 10x Genomics linked-read technology. In addition, we analyzed the expression level dominance and homoeologous expression bias in the allotetraploid *V. reflexo-pilosa*. These genome assemblies along with their annotation provide valuable resources for *Vigna* breeding programs, and the knowledge on duplicated gene expression and epigenetic alterations in *V. reflexo-pilosa* provides new insight that enhances our understanding of the allopolyploidization process.

## Materials and Methods

### Plant materials and DNA/RNA isolation

For whole genome sequencing, young leaf tissues were collected from 5-week-old plants (*V. reflexo-pilosa* accession: AusTRCF30263, *V. hirtella* accession: JP226635 and *V. trinervia* accession: J226670; Supplementary Figure 1), immediately frozen and stored in liquid nitrogen until use. DNA was isolated following the protocol in (Pootakham et al., 2022). Briefly, the frozen tissue was ground in liquid nitrogen and CTAB buffer was added. DNA was extracted from the aqueous phase using 25:24:1 phenol:chloroform:isoamyl alcohol and precipitated in 100% ethanol. DNA pellets were washed twice with 70% ethanol, air-dried and resuspended in 10 mM Tris-HCl pH 8.0. After purification with the Ampure PB beads (Pacific Biosciences, Menlo Park, USA), DNA integrity was evaluated using Pippin Pulse Electrophoresis System (Sage Science, Beverly, USA). For transcriptome sequencing, leaves, roots, flowers, 1-week-old pods and 3-week-old pods were collected from the same individuals used for whole genome sequencing, snap-frozen and stored in liquid nitrogen until use. Total RNA was extracted using the CTAB buffer and 25:24:1 phenol:chloroform:isoamyl alcohol and precipitated overnight in ¼ vol 8M LiCl. RNA pellets were washed twice with 70% ethanol, air-dried and resuspended in RNase-free water. Poly(A) mRNA was enriched from total RNA using the Dynabeads mRNA Purification Kit (ThermoFisher Scientific, Waltham, USA). The Fragment Analyzer system (Agilent, Santa Clara, USA) was used to evaluate the integrity of RNA samples.

### DNA and RNA sequencing library preparation

For *V. reflexo-pilosa* reference genome sequencing, a PacBio SMRTbell library with an insert size of 10,000 nt was prepared from the high molecular weight DNA template using SMRTbell Express Template Prep Kit 2.0 and sequenced on the PacBio Sequel system (Pacific Biosciences, Menlo Park, USA). For *V. hirtella* and *V. trinervia* whole genome sequencing, the 10x Genomics libraries were prepared from 1.25 ng of high molecular weight DNA using the Chromium Genome Library Kit and Gel Bead Kit v2, the Chromium Genome Chip Kit v2 and the Chromium i7 Multiplex Kit following the manufacturer’s instructions (10x Genomics, Pleasanton, USA). The 10x Genomics libraries were sequenced on the Illumina HiSeq X Ten (PE150).

For transcriptome sequencing, Iso-seq libraries were prepared using the NEBNext Single Cell/Low Input cDNA Synthesis and Amplification Module (New England Biolabs, Ipswich, USA), Iso-Seq Express Oligo Kit and SMRTbell Express Template Prep Kit 2.0 (Pacific Biosciences, Menlo Park, USA). Sequencing was performed with the Sequel Binding Kit 2.0 using a 20-h movie collection time following the manufacturer's protocol (Pacific Biosciences, Menlo Park, USA). To obtain short-read RNA sequences, 200 ng of poly(A) mRNA was used to construct libraries using the MGIEasy RNA Library Prep Kit V3.0 (MGI Tech, China). The libraries were sequenced on the MGISEQ-2000RS using the MGISEQ-2000RS Sequencing Flow Cell v3.0 (MGI Tech, China).

**Hi-C library preparation and sequencing**

A chromosome conformation capturing technique (Hi-C) was conducted by Dovetail Genomics (Scott Valley, USA) to scaffold *V. reflexo-pilosa* preliminary assembly into a chromosome-level assembly. A Hi-C library was prepared as previously described in (Lieberman-Aiden et al., 2009). Briefly, chromatin was fixed in place with formaldehyde in the nucleus and then extracted fixed chromatin was digested with *DpnII*, the 5' overhangs filled in with biotinylated nucleotides, and then free blunt ends were ligated. After ligation, crosslinks were reversed and the DNA purified from protein. Purified DNA was treated to remove biotin that was not internal to ligated fragments. The DNA was then sheared to ~350 bp mean fragment size and sequencing libraries were generated using NEBNext Ultra enzymes and Illumina-compatible adapters. Biotin-containing fragments were isolated using streptavidin beads before PCR enrichment of each library. The libraries were sequenced on an Illumina HiSeq X to produce 50,357,334 read pairs.

***De novo* assembly**

For *V. reflexo-pilosa*, a total of 6,496,297 PacBio raw reads totaling 69.38 Gb were subjected to read correction, trimming, overlap detection and de novo assembly by Canu v1.9 (Koren et al., 2017) using the following parameters: genomeSize = 900m, correctedErrorRate = 0.040. For other parameters, default settings were used. An estimated genome size of 900 Mb was assumed according to our flow cytometry results (see the 'Results' section below). The polishing was carried out using the GenomicConcensus package in PacBio® tools distributed via

Bioconda (https://github.com/PacificBiosciences/pbbioconda). The PacBio draft assembly was used as an input for the subsequent scaffolding with HiRise, a software pipeline designed specifically for using proximity ligation data to scaffold genome assemblies (Putnam et al., 2016). For *V. hirtella* and *V. trinervia*, the Supernova assembler version 2.1.1 was used to assemble linked-read data using the default settings (10x Genomics, Pleasonton, USA).

**Hi-C scaffolding of the *V. reflexo-pilosa* genome**

The input *de novo* PacBio assembly and Dovetail Hi-C library reads were used as input data for HiRise. Dovetail Hi-C library sequences were aligned to the draft input assembly using bwa (https://github.com/lh3/bwa). The separations of Dovetail Hi-C read pairs mapped within draft scaffolds were analyzed by HiRise to produce a likelihood model for genomic distance between read pairs, and the model was used to identify and break putative misjoins, to score prospective joins, and make joins above a threshold.

**Genome assembly evaluation**

The quality of the final genome assembly was evaluated by aligning short-read DNA sequences (10x Genomics data) and transcriptome (Iso-seq or RNA-seq) data from this study using BWA version 0.7.17-r1188 for DNA sequence alignment, minimap version 2.17 for Iso-seq alignment and HISAT2 version 2.2.0 for RNA alignment. In addition, the presence and completeness of the orthologs were determined using the Benchmarking Universal Single-Copy Orthologues (BUSCO) version 4.0.5 (Simão et al., 2015) and the Embryophyta OrthoDB release 10 (Kriventseva et al., 2015). PolyCRACKER software was employed to separate two subgenomes in the *V. reflexo-pilosa* assembly using signatures of repetitive DNA evolution (Gordon et al., 2019).

**Genome size estimation**

To estimate the nuclear DNA content using flow cytometry, fresh leaf tissues from *V. reflexo-pilosa, V. hirtella* and *V. trinervia* were cut into small pieces with a sharp razor blade and analyzed using the protocol in (Dolezel and Bartos, 2005). We used the Galbraith's buffer reported in (Galbraith et al., 1983) as a nuclear isolation buffer. Nuclei were stained with 50 ug/mL of propidium iodide (Thermo Fisher Scientific, Waltham, USA). Maize (*Zea mays*) was used as the DNA reference standard.

**Identification of repetitive sequences and gene annotation**

To identify repetitive element families in the genome assembly, RepeatModeler version 2.0.3 (http://www.repeatmasker.org/RepeatModeler/) was used to construct a *de novo* repeat library. This pipeline employed two distinct repeat discovery algorithms, RECON (version 1.08) and RepeatScout (version 1.0.6), to identify the boundaries of repetitive elements and build consensus models of interspersed repeats (Bao and Eddy, 2002;Price et al., 2005). We aligned repeat sequences in the library to GenBank's nr protein database using BLASTX (e-value cutoff = $10^{-6}$) to ensure that they did not contain large families of protein-coding sequences.

To identify protein-coding sequences in the unmasked assembly, we used EvidenceModeler (EVM) version 1.1.1 to combine evidences from RNA-based prediction, homology-based prediction and *ab initio* prediction (Haas et al., 2008). For RNA-based prediction, we used evidence from PacBio Iso-seq data obtained from leaf, stem and flower tissues. Full-length transcripts were mapped to the final assembly using the genomic mapping and alignment program (GMAP) version 2020-09-12 (Wu and Watanabe, 2005). Protein sequences from *Glycine max, Phaseolus vulgaris, Vigna unguiculata, Vigna angularis, Vigna mungo* and *Arabidopsis thaliana* available on the public databases were aligned to the unmasked genome using AAT (Huang et al., 1997). Protein-coding gene predictions were obtained with Augustus version 3.2.1 (Stanke et al., 2004) trained with *G. max, P. vulgaris, V. unguiculata, V. angularis, V. mungo, V. reflexo-pilosa* and *A. thaliana* PASA transcriptome alignment assembly using *V. reflexo-pilosa* alignment files as inputs. All gene predictions were integrated by EVM to generate consensus gene models using the following weights for each evidence type: PASA2 – 1, GMAP – 0.5, AAT – 0.3 and Augustus – 0.3. Any predicted genes that had more than 20% overlapping sequence with repetitive sequences or had no RNA-seq support were excluded from the list of annotated genes.

**Phylogenetic analyses and comparative genomics**

OrthoFinder version 2.4.0 (Emms and Kelly, 2019) was used to identify orthologous groups in *A. thaliana, Citrullus lanatus, Cucumis melo, Cucumis sativus, G. max, Oryza sativa, P. vulgaris, V. hirtella, V. mungo, Vigna radiata, V. reflexo-pilosa, V. trinervia*

*and V. unguiculata.* We constructed a phylogenetic tree based on protein sequences from single-copy orthologous groups using RAxML-NG program version 1.0.2 (Stamatakis, 2006). We first aligned protein sequences in each single-copy orthologous group with MUSCLE (Edgar, 2004) and removed alignment gaps with trimAI version 1.4 rev15 (Capella-Gutiérrez et al., 2009) using the automated1 heuristic method. We subsequently concatenated alignment blocks using catsequences program (https:// github.com/ChrisCreevey/catsequences), and the substitution model for each block was estimated using the ModelTest-NG program version 0.1.7 (Darriba et al., 2020). The outputs were used to compute a maximum-likelihood phylogenetic tree. Divergence times were estimated using the MCMCtree software version 4.0 (PAML 4 package) (Yang, 2007) using the relaxed-clock model with the known divergence time between *C. melo* and *C. sativus*, which was estimated at 8.4–11.8 million years ago (MYA) (Sebastian et al., 2010).

**Genome synteny analysis**

MCscanX (Wang et al., 2012) was used to analyze the colinearity within the *V. reflexo-pilosa* genome and between *V. reflexo-pilosa – G. max, V. reflexo-pilosa – P. vulgaris, V. reflexo-pilosa – V. hirtella, V. reflexo-pilosa – V. mungo, V. reflexo-pilosa – V. radiata, V. reflexo-pilosa – V. trinervia and V. reflexo-pilosa – V. unguiculata* genomes. *V. reflexo-pilosa* amino acid sequences were aligned against themselves, *G. max, P. vulgaris, V. hirtella, V. mungo, V. radiata, V. trinervia* or *V. unguiculata* using BLASTP (with an e-value cutoff of $10^{-10}$) in order to identify putative paralogs. Intragenic homeologous blocks were defined as regions of ten or more genes with colinear or nearly colinear runs of paralogs elsewhere in the genome with fewer than six intervening genes. These intragenic homeologous blocks were visualized using CIRCOS version 0.69.8 (Krzywinski et al., 2009). Similarly, we also performed pairwise comparisons of input protein sequences from *V. reflexo-pilosa*, *V. mungo* and *V. radiata*. Clustering was carried out using OrthoMCL software version 2.0.9 (Li et al., 2003) based on a Markov clustering algorithm (MCL). Syntenic blocks between *V. reflexo-pilosa*, *V. mungo* and *V. radiata* were identified by MCscanX and plotted with CIRCOS using the criteria mentioned above (at least ten colinear genes and fewer than six intervening genes allowed).

**Expression level dominance and homoeolog expression bias analyses**

To investigate the changes in expression level dominance and homoeolog expression bias in the tetraploid *V. reflexo-pilosa*, we compared the expression levels of 15,597 *V. hirtella*-*V. trinervia* orthologous gene pairs following the method reported in (Wu et al., 2018). Twelve possible profiles of gene expression, including additivity, expression level dominance and transgressive were classified according to (Yoo et al., 2012). For the homoeolog expression bias analysis, we compared the expression level of each homoeolog pair in the diploid progenitors (*V. hirtella* and *V. trinervia*) and the tetraploid *V. reflexo-pilosa* using the Student's *t*-test ($P \leq 0.05$) according to the method reported in a previous study (Wu et al., 2018).

**DNA methylation analysis**

Whole genome bisulfite sequencing was carried out in *V. reflexo-pilosa*, *V. hirtella* and *V. trinervia* on the Illumina HiSeq X Ten (PE150) by Omics Drive Pte Ltd. (Singapore). We utilized Batmeth2 (https://github.com/GuoliangLi-HZAU/BatMeth2), an integrated multi-functional software for DNA methylation analysis, which includes DNA methylation level computation, DNA methylation sequence alignment and functional annotation (Zhou et al., 2019). Chloroplast sequences (which are fully unmethylated in plants) were used as internal controls to calculate the sodium bisulfite reaction nonconversion rate of unmodified cytosines. Genome-wide DNA methylation levels were calculated for all three contexts (CG, CHG and CHH) using the weighted methylation level (Schultz et al., 2012). For metaplots, cytosines from 2,000 bases upstream and downstream and within coding region and transposable element (TE) bodies were extracted. These regions (2-kb upstream, gene/TE body, 2-kb downstream) were divided into 20 bins, and weighted methylation levels were computed for each bin according to the protocol previously described in (Schultz et al., 2012).

## Results

### Genome assembly of *V. reflexo-pilosa*, *V. hirtella* and *V. trinervia*

To obtain the whole genome sequences of the tetraploid hybrid *V. reflexo-pilosa* and its progenitors, *V. hirtella* and *V. trinervia*, we employed two sequencing technologies: the long-read PacBio sequencing for *V. reflexo-pilosa* (AusTRCF30263) and the linked-read 10x Genomics sequencing for *V. hirtella* (JP226635) and *V. trinervia* (J226670). For *V. reflexo-pilosa*, we generated a total of 6,496,297 raw reads (69.38 Gb) representing ~65X coverage based on the estimated genome size of 1.07 Gb obtained from DNA flow cytometry (Supplementary Figure 2). A *de novo* assembly of PacBio sequences yielded a draft genome of 985,902,075 Mb in 3,314 contigs with a contig N50 of 1,851,115 (L50 = 161 contigs; Table 1). This preliminary assembly was further scaffolded using the *in vivo* chromosome fixation technique (Hi-C) based on the information from 50,357,334 read pairs. The final assembly contained 22 chromosome-level pseudomolecules greater than 10 Mb (hereafter referred to as chromosomes numbered according to sizes; Figure 1). These 22 chromosomes covered 923,741,909 bases or 92.55% of the 998-Mb *V. reflexo-pilosa* assembly.

For *V. hirtella* and *V. trinervia* whole genome sequencing, we produced a total of 100.15 Gb and 108.36 Gb of Illumina paired-end 150 bp sequencing data from 667,706,838 and 722,446,750 raw reads, respectively. These reads represented ~194X and ~214X coverage based on the estimated genome sizes of 516 Mb (*V. hirtella*) and 506 Mb (*V. trinervia*), respectively, obtained by DNA flow cytometry (Supplementary Figure 2). We performed *de novo* assembly of linked-read sequences using the Supernova, generating draft genomes of 474 Mb (N50 = 2,078,642 bases) and 498 Mb (N50 = 1,656,192 bases) for *V. hirtella* and *V. trinervia*, respectively (Table 1).

We evaluated the quality of our assemblies by aligning short-read genomic sequence data to the genomes. Over 98% of the DNA short read sequences from *V. hirtella* and *V. trinervia* could be mapped to their respective genomes, with the mapped rates of 98.4% and 98.1%, respectively. We also mapped Iso-seq and RNA-seq reads to their respective genomes, and 99.2% of *V. reflexo-pilosa* Iso-seq transcripts were mapped to the genome while 91.4%, 90.4% and 91.8% of *V. reflexo-*

*pilosa, V. hirtella* and *V. trivervia* RNA-seq reads could be aligned to the respective genomes. To further assess the completeness of the gene space in each assembly, we used the BUSCO software to check the gene content using a plant-specific database of 1,440 genes (Simão et al., 2015). In *V. reflexo-pilosa*, our gene prediction recovered 97.5% of the highly conserved orthologs in the Embryophyta lineage with 11.0% identified as "complete and single-copy," 85.7% as "complete and duplicated" and 0.8% as "fragmented" while 2.5% of the conserved orthologs were missing from the assembly (Table 1). The gene predictions for *V. hirtella* and *V. trinervia* recovered 99.0% (complete and single-copy: 94.1%, complete and duplicated: 3.3%, fragmented: 1.6%) and 98.0% (complete and single-copy: 91.6%, complete and duplicated: 2.9%, fragmented: 3.5%) of the highly conserved orthologs, respectively (Table 1).

**Gene annotation**

We integrated three different approaches including an *ab initio* prediction, a homology-based search and a transcript-based prediction in the annotation pipeline to predict 68,448, 38,124 and 35,231 gene models and 59,382, 34,371 and 31,017 protein-coding genes in *V. reflexo-pilosa*, *V. hirtella* and *V. trinervia*, respectively. Coding sequences in *V. reflexo-pilosa* were preferentially distributed near the telomeres for most of the chromosomes (Figure 1). In all three *Vigna* species, the most prevalent gene ontology (GO) terms associated with cellular component were integral component of membrane and nucleus (Supplementary Figures 3-5). The largest categories of genes annotated to molecular function were ATP binding and metal binding whereas the most common terms for biological process were protein phosphorylation and regulation of transcription (Supplementary Figures 3-5).

**Identification of repetitive elements in the *Vigna* genomes**

We employed a combination of a *de novo* repeat identification tool, RepeatModeler, and homology search tools to analyze repetitive sequences in three *Vigna* species studied here. The total length of repetitive sequences in *V. reflexo-pilosa* accounted for 44.4% of the assembly, slightly higher than the proportions of repetitive sequences observed in *V. hirtella* (41.0%) and *V. trinervia* (38.7%; Table 2). The total bases covered by the repetitive sequences in *V. reflexo-pilosa* assembly (438 Mb) were slightly higher than the sum of the repetitive regions identified in the two

progenitors, *V. hirtella* (194 Mb) and *V. trinervia* (192 Mb). Retrotransposons occupied significant portions of the repetitive elements in all three genomes, comprising 26.0%, 22.2% and 22.3% of the *V. reflexo-pilosa, V. hirtella* and *V. trinervia* assemblies, respectively. Of the retrotransposons, *Copia* and *Gypsy* represented the majority of the long terminal repeats (LTRs) in all three *Vigna* species examined (Table 2).

**Comparative genomics and phylogenetic analyses**

To determine the phylogenetic relationship between *V. reflexo-pilosa* and other plant species, we analyzed sequence information from single-copy orthologous genes from eight legumes: *G. max* (soybean), *P. vulgaris* (common bean), *V. hirtella*, *V. mungo* (black gram), *V. radiate* (mungbean), *V. reflexo-pilosa*, *V. trinervia* and *V. unguiculata* (cowpea); three cucurbit species: *C. lanatus* (watermelon), *C. melo* (melon), *C. sativus* (cucumber); one rosid species (*A. thaliana*) and one monocot representative *(O. sativa*; rice) included as an outgroup. Cucumber and melon were included in the analysis because of their known divergence time (Sebastian et al., 2010). A maximum-likelihood phylogenetic tree obtained from clustering 490,264 proteins into 36,444 orthologous groups (516,828 input proteins from 13 species) suggested that *V. reflexo-pilosa* had a close relationship with its progenitors, *V. hirtella* and *V. trinervia*, as well as *V. mungo* and *V. radiata*. The phylogenetic tree also revealed that *V. reflexo-pilosa* and *V. hirtella* diverged approximately 5.09 MYA, and their ancestor diverged from the last common ancestor of *V. trinervia*, *V. mungo* and *V. radiata* roughly 5.47 MYA (Figure 2).

We analyzed orthologous gene pairs in order to estimate relative timing of divergence between *V. reflexo-pilosa* and related species using the 4DTv method, which measured the number of transversions at fourfold degenerate synonymous sites. The peak 4DTv distances between *V. reflexo-pilosa* – *V. radiata* (0.016) and *V. reflexo-pilosa* – *V. mungo* (0.017) were smaller than the peak 4DTv distances between *V. reflexo-pilosa* and *V. unguiculata* (0.037), *P. vulgaris* (0.054) and *G. max* (0.102), suggesting that *V. reflexo-pilosa* diverged from *V. unguiculata, P. vulgaris* and *G. max* before the speciation event separating *V. reflexo-pilosa, V. radiata* and *V. mungo* (Figure 2). We compared 33,010 paralogous gene pairs residing within duplicated collinear blocks in *V. reflexo-pilosa* assembly and observed two peaks at

0.021 and 0.241, suggesting that créole bean has experienced a recent whole genome duplication event. This is in accordance with the presence of intragenomic synteny blocks throughout the genome (Figure 1). Examination of synteny between *V. reflexo-pilosa* and *V. radiata* and *V. mungo* also supported the occurrence of a genome-wide duplication in *V. reflexo-pilosa* (Supplementary Figure 6).

**Analyses of homoeolog expression bias and expression level dominance in the tetraploid *V. reflexo-pilosa***

In allotetraploid species, duplicated gene pairs may show preferential expression of one homoeolog relative to the other, and the term used to describe the differences is homoeolog expression bias (Grover et al., 2012). Homoeolog expression bias has been documented in several allopolyploids, including *Brassica* (Wu et al., 2018), *Gossypium* (Hovav et al., 2008;Nettleton et al., 2008), *Coffea* (Combes et al., 2011) and *Triticum* (Powell et al., 2016). To examine the homoeolog expression bias in the tetraploid *V. reflexo-pilosa*, we monitored the expression levels of 15,597 homoeologous gene pairs. Nearly two thirds (65.53%) of all expressed homoeolog pairs in *V. reflexo-pilosa* were maintained in the parental condition while 14.29% of homoeolog pairs with pre-existing expression bias in the diploid parents reverted to non-differential expression in the allotetraploid (Figure 3). Interestingly, 20.17% of the homoeolog pairs displayed novel bias in the progeny. Overall, the majority of the homoeolog pairs (11,374 out of 15,597 or 72.92%) exhibited no expression bias in *V. reflexo-pilosa*, and the remaining (4,223 homoeolog pairs; 27.08%) showed biased expression. The allotetraploid *V. reflexo-pilosa* displayed unbalanced homoeolog expression bias with a preference toward the *V. trinervia* subgenome (16.33% = *V. trinervia*-biased and 10.74% = *V. hirtella*-biased; Figure 3).

In addition to homoeolog expression bias in duplicated gene pairs, expression level dominance has also been observed in allopolyploid species (Rapp et al., 2009;Grover et al., 2012). Expression level dominance does not consider relative expression levels of individual homoeologs but refers to the total expression of a duplicated gene pair compared to its progenitors. Genes identified as differentially expressed in the allotetraploid relative to their diploid progenitors were categorized into 12 possible expression groups (Figure 3) including additivity (I and XII), *V. hirtella*-expression level dominance (II and XI), *V. trinervia*-expression level

dominance (IV and IX), transgressive expression lower than either parent (III, VII and X) and transgressive expression higher than either parent (V, VI and VIII). Overall, 22.17% of the homoeolog pairs in the tetraploid *V. reflexo-pilosa* exhibited no change in the expression (i.e. the total expression level of a duplicated gene pair was equal to that in both diploid parents) (Figure 3). Over a third (36.24%) of homoeolog pairs displayed expression level dominance with significantly more *V. trinervia*-expression level dominance (33.49%, categories IV and IX) than *V. hirtella*-expression level dominance (2.75%, categories II and XI). The gene expression in *V. reflexo-pilosa* appeared to be bias toward the *V. trinervia* subgenome. Notably, the largest class of expression level observed in *V. reflexo-pilosa* was transgressive expression (41.24%). Almost all transgressive regulation homoeolog pairs were transgressive up-regulation (41.21% transgressive up-regulation, categories V, VI and VIII; 0.03% transgressive down-regulation, categories III, VII and X; Figure 4). Very few gene pairs (0.35%, categories I and XII) exhibited additivity expression pattern.

**DNA methylation patterns in parents and allotetraploid**

To understand how DNA methylation pattern changed following polyploidization, we investigated the methylation status in parents (*V. hirtella* and *V. trinervia*) and the allotetraploid (*V. reflexo-pilosa*) at CG, CHG (where H = C, A, T) and CHH sites using whole-genome bisulfite sequencing. Methylation patterns in genes and TEs and their upstream and downstream regions were compared. In coding regions, CHG and CHH methylation patterns are similar in the tetraploid and *V. hirtella* parents while the CG methylation level in *V. reflexo-pilosa* was intermediate between the two donor species (Figure 5A). For TEs, CG and CHH methylation profiles in *V. reflexo-pilosa* and *V. hirtella* were almost identical whereas the CHG methylation level in *V. reflexo-pilosa* was noticeably lower than that in *V. trinervia* (Figure 5B).

When analyzed by subgenomes, methylation levels in the coding regions were on average lower for the *V. trinervia* subgenome (in *V. reflexo-pilosa*; VRt) compared to the *V. trinervia* (VT) parental genome for every methylation context (Figure 6). A similar trend was observed for coding regions in the *V. hirtella* subgenome (in *V. reflexo-pilosa*; VRh) and *V. hirtella* (VH) parental genome for CG and CHG methylation. The level of CHH methylation in coding regions was relatively similar for the VRh subgenome and *V. hirtella.* For TEs, the levels of methylation in

the VRh subgenome essentially mirrored those in *V. hirtella* in all three contexts (Figure 6). While the levels of CG and CHG methylation in TE bodies were nearly identical between the VRt subgenome and *V. trinervia* genome, the level of CHH methylation was much lower in the VRt subgenome than that in *V. trinervia*.

**Conclusion**

We reported the first high-quality reference genome assembly of the neglected crop, *V. reflexo-pilosa*, the only tetraploid species in genus *Vigna*. We employed the long-read PacBio sequencing and the Hi-C chromatin contact mapping to achieve the chromosome-scale assembly of the créole bean genome. We also sequenced and assembled the genomes of its donor species, *V. hirtella* and *V. trinervia*. Comparative genomic and phylogenetic analyses revealed that *V. reflexo-pilosa* and *V. hirtella* diverged approximately 5.09 MYA, and their ancestor diverged from the last common ancestor of *V. trinervia*, *V. mungo* and *V. radiata* roughly 5.47 MYA. We also examined gene expression bias and differences in methylation levels between two progenitor genomes. The allotetraploid *V. reflexo-pilosa* displayed unbalanced homoeolog expression bias with a preference toward the *V. trinervia* subgenome, and one third of the homoeolog pairs (33.49%) in *V. reflexo-pilosa* also displayed VT-expression level dominance. The average CG, CHG and CHH methylation levels in coding regions were higher in the VRh subgenome than those in the VRt subgenome, in concordance with the observed homoeolog expression bias toward the *V. trinervia* subgenome. Our high-quality genome assemblies provide valuable resources for accelerating *Vigna* breeding programs, studying comparative genomics/phylogenetics and investigating genetic and epigenetic changes following hybridization and polyploidization events.

**Data Availability**

*V. reflexo-pilosa* genome assembly and transcriptome data have been submitted to the DDBJ/EMBL/GenBank databases under Bioproject PRJNA705823 and the following accession numbers: JAFNIE000000000 (genome assembly), SRR22875419 (bisulfite sequence data), SRR23057277 (Iso-seq; leaf), SRR23057275 (Iso-seq; pod),

SRR23057274 (Iso-seq; root), SRR23057276 (Iso-seq; flower) and SRR23057273 (RNA-seq). *V. trinervia* genome assembly and transcriptome data have been submitted to the DDBJ/EMBL/GenBank databases under Bioproject PRJNA717125 and the following accession numbers: JAJDEZ000000000 (genome assembly), SRR22875418 (bisulfite sequence data) and SRR23086091 (RNA-seq). *V. hirtella* genome assembly and transcriptome data have been submitted to the DDBJ/EMBL/GenBank databases under Bioproject PRJNA716777 and the following accession numbers: JAHCHA000000000 (genome assembly), SRR22875417 (bisulfite sequence data) and SRR23104076 (RNA-seq).

**Funding**

This work was supported by the National Science and Technology Development Agency (NSTDA), Grant number 1000221.

**Competing Interests**

The authors declare that the research described herein was conducted in the absence of any commercial or financial relationships that could be construed as potential competing interests.

**Author contributions**

Research study was designed by W.P. and S.T. Sample collection and laboratory work (DNA/RNA extraction, sequencing library preparation, flow cytometry) were performed by W.P., C.Y., T.Y., D.S. and P.S. Bioinformatics analyses were performed by W.K., C.N. and C.S. The manuscript was written and revised by W.P. and all authors reviewed it.

**Tables**

**Table 1.** Assembly statistics of *V. reflexo-pilosa*, *V. hirtella* and *V. trinervia* genomes.

| | ***V. reflexo-pilosa*** | | ***V. hirtella* 10x Genomics** | ***V. trinervia* 10x Genomics** |
|---|---|---|---|---|
| | **PacBio** | **PacBio + HiC** | | |
| N50 scaffold size (bases) | 1,851,115 | 42,545,650 | 2,078,642 | 1,656,192 |
| L50 scaffold number | 161 | 10 | 64 | 83 |
| N75 scaffold size (bases) | 974,853 | 34,137,080 | 681,869 | 518,280 |
| L75 scaffold number | 344 | 17 | 162 | 211 |
| N90 scaffold size (bases) | 294,338 | 31,159,791 | 12,396 | 12,517 |
| L90 scaffold number | 598 | 21 | 1,331 | 2,425 |
| Assembly size (bases) | 985,902,075 | 998,724,930 | 474,134,210 | 498,783,358 |
| Number of scaffolds | 3,314 | 2,660 | 17,238 | 17,328 |
| Number of scaffolds ≥ 100 kb | 765 | 46 | 314 | 329 |
| Number of scaffolds ≥ 1 Mb | 337 | 22 | 124 | 142 |
| Number of scaffolds ≥ 10 Mb | 0 | 22 | 0 | 0 |
| Longest scaffold (bases) | 8,094,978 | 73,324,288 | 8,749,190 | 9,277,022 |
| % N | 0 | 0.05 | 5.27 | 12.6 |
| GC content (%) | 33.99 | 33.93 | 33.7 | 33.33 |
| BUSCO evaluation (% completeness) | 97.3 | 97.5 | 99.0 | 98.0 |
| complete and single-copy: | 10.8 | 11.0 | 94.1 | 91.6 |
| complete and duplicated: | 85.4 | 85.7 | 3.3 | 2.9 |
| fragmented: | 1.1 | 0.8 | 1.6 | 3.5 |
| missing: | 2.7 | 2.5 | 1.0 | 2.0 |

**Table 2.** Repeat contents in *V. reflexo-pilosa*, *V. hirtella* and *V. trinervia* genomes.

| Types of repeats | Bases (Mb) | % of the assembly | % of total repeats |
|---|---|---|---|
| ***V. reflexo-pilosa*:** | | | |
| DNA transposons | 50.57 | 5.13 | 11.53 |
| Retrotransposons | | | |
| LINE | 5.70 | 0.57 | 1.30 |
| SINE | 0.03 | 0.03 | 0.07 |
| LTR: *Copia* | 92.79 | 9.42 | 21.16 |
| LTR: *Gypsy* | 155.32 | 15.74 | 35.43 |
| LTR: Others | 2.9 | 0.29 | 0.66 |
| Simple sequence repeats | 29.96 | 3.04 | 6.83 |
| Others | 101.1 | 10.22 | 23.32 |
| Total | 438.37 | 44.44 | |
| ***V. hirtella*:** | | | |
| DNA transposons | 25.07 | 5.28 | 12.88 |
| Retrotransposons | | | |
| LINE | 1.34 | 0.28 | 0.68 |
| SINE | 0.001 | 0.003 | 0.007 |
| LTR: Copia | 38.20 | 8.05 | 19.63 |
| LTR: Gypsy | 64.96 | 13.70 | 33.38 |
| LTR: Others | 1.02 | 0.21 | 0.52 |
| Simple sequence repeats | 8.89 | 1.87 | 4.56 |
| Others | 54.97 | 11.64 | 28.34 |
| Total | 194.45 | 41.03 | |
| ***V. trinervia*:** | | | |
| DNA transposons | 21.0 | 4.21 | 10.89 |
| Retrotransposons | | | |
| LINE | 1.35 | 0.27 | 0.70 |
| SINE | 0.009 | 0.018 | 0.046 |
| LTR: Copia | 50.13 | 10.05 | 25.99 |
| LTR: Gypsy | 58.50 | 11.72 | 30.34 |
| LTR: Others | 1.44 | 0.28 | 0.74 |
| Simple sequence repeats | 8.66 | 1.73 | 4.49 |
| Others | 51.72 | 10.38 | 26.80 |
| Total | 192.81 | 38.66 | |

**Figure legends**

**Figure 1.** Genomic landscape of *V. reflexo-pilosa.* (I) A physical map of 22 pseudomolecules (chromosomes) numbered according to size (in Mb) (II) Subgenome assignment using PolyCRACKER: yellow and green colors indicate sequences belonging to *V. hirtella* and *V. trinervia*, respectively (III) Repeat content represented by the proportion of genomic regions covered by repetitive sequences in 250-kb windows (IV) Gene density represented by the number of genes in 250-kb windows (V) GC content represented by the percentage of G + C bases in 250-kb windows. Syntenic blocks in the genome are displayed by connected lines.

**Figure 2.** Comparative genomics of *V. reflexo-pilosa* and related species. (A) A maximum-likelihood tree of *V. reflexo-pilosa*, five *Vigna* species and seven other plant species constructed based on single-copy orthologous protein sequences. Numbers at each node represent the estimated divergence time in million years ago (MYA). (B) The distribution of fourfold synonymous third-codon transversion position (4DTv) distances between paralogous genes in *V. reflexo-pilosa*, *V. hirtella*, *V. trinervia*, *V. mungo*, *V. radiata*, *V. unguiculata*, *G. max* and *P. vulgaris*. (C) The distribution of 4DTv distances between orthologous genes in *V. reflexo-pilosa* and *V. hirtella*, *V. trinervia*, *V. mungo*, *V. radiata*, *V. unguiculata*, *G. max* and *P. vulgaris*. Peaks of intraspecific and interspecific 4DTv distributions indicate whole genome duplication and speciation events, respectively.

**Figure 3.** Homoeologous expression bias in the allotetraploid *V. reflexo-pilosa*. Sizes of the circles represent the relative expression levels of the homoeologs in donor species, *V. hirtella* and *V. trinervia*. The area ratios of the circles in the tetraploid *V. reflexo-pilosa* represent the relative expression levels of the homoeologs. The number of homoeolog pairs and the percentage of the total number of expressed homoeolog pairs analyzed are shown for each condition. VH, VT, VRh and VRt indicate *V. hirtella* parental genome, *V. trinervia* parental genome, *V. hirtella* subgenome of *V. reflexo-pilosa* and *V. trinervia* subgenome of *V. reflexo-pilosa*, respectively.

**Figure 4.** Expression level dominance (ELD) in the allotetraploid *V. reflexo-pilosa*. The number (and percentage) of homeologous gene pairs exhibiting additive expression,

transgressive expression and ELD are indicated. VH, VR, VT denote *V. hirtella, V. reflexo-pilosa* and *V. trinervia,* respectively.

**Figure 5.** DNA methylation of (A) genes and (B) TEs in the allotetraploid *V. reflexo-pilosa* and its parental species. Metaplots of CG, CHG and CHH mean weighted methylation of annotated genes evaluated using bisulfite sequencing in *V. reflexo-pilosa* (blue; VR), *V. hirtella* (red; VH) and *V. trinervia* (green; VT). Methylation levels are displayed for coding regions, transcription start site (TSS), transcription end site (TES) and 2,000 bases upstream and downstream of the TSS and TES.

**Figure 6.** Subgenome-specific methylation in the allotetraploid. Weighted methylation levels of *V. hirtella* and *V. trinervia* subgenome-specific patterns in genes (A, C) and TEs (B, D). Methylation levels of each individual are indicated as follows: *V. hirtella* parent (VH) = red, *V. trinervia* parent (VT) = green, *V. hirtella* subgenome (in *V. reflexo-pilosa*; VRh) = dark blue and *V. trinervia* subgenome (in *V. reflexo-pilosa*; VRt) = light blue. Methylation levels are displayed for coding regions, transcription start site (TSS), transcription end site (TES) and 2,000 bases upstream and downstream of the TSS and TES.

## References

Bao, Z., and Eddy, S.R. (2002). Automated de novo identification of repeat sequence families in sequenced genomes. *Genome research* 12**,** 1269-1276.

Capella-Gutiérrez, S., Silla-Martínez, J.M., and Gabaldón, T. (2009). trimAl: a tool for automated alignment trimming in large-scale phylogenetic analyses. *Bioinformatics* 25**,** 1972-1973.

Chankaew, S., Isemura, T., Isobe, S., Kaga, A., Tomooka, N., Somta, P., Hirakawa, H., Shirasawa, K., Vaughan, D.A., and Srinives, P. (2014). Detection of Genome Donor Species of Neglected Tetraploid Crop Vigna reflexo-pilosa (Créole Bean), and Genetic Structure of Diploid Species Based on Newly Developed EST-SSR Markers from Azuki Bean (Vigna angularis). *PLOS ONE* 9**,** e104990. doi: 10.1371/journal.pone.0104990.

Chen, Z.J. (2007). Genetic and epigenetic mechanisms for gene expression and phenotypic variation in plant polyploids. *Annu. Rev. Plant Biol.* 58**,** 377-406.

Combes, M.-C., Cenci, A., Baraille, H., Bertrand, B., and Lashermes, P. (2011). Homeologous Gene Expression in Response to Growing Temperature in a Recent Allopolyploid (Coffea arabica L.). *Journal of Heredity* 103**,** 36-46. doi: 10.1093/jhered/esr120.

Darriba, D., Posada, D., Kozlov, A.M., Stamatakis, A., Morel, B., and Flouri, T. (2020). ModelTest-NG: a new and scalable tool for the selection of DNA and protein evolutionary models. *Molecular Biology and Evolution* 37**,** 291-294.

Dolezel, J., and Bartos, J. (2005). Plant DNA flow cytometry and estimation of nuclear genome size. *Annals of Botany* 95**,** 99-110. doi: 10.1093/aob/mci005.

Doyle, J.J., Flagel, L.E., Paterson, A.H., Rapp, R.A., Soltis, D.E., Soltis, P.S., and Wendel, J.F. (2008). Evolutionary genetics of genome merger and doubling in plants. *Annual review of genetics* 42**,** 443-461.

Edgar, R.C. (2004). MUSCLE: multiple sequence alignment with high accuracy and high throughput. *Nucleic Acids Res* 32**,** 1792-1797. doi: 10.1093/nar/gkh340.

Edger, P.P., Poorten, T.J., Vanburen, R., Hardigan, M.A., Colle, M., Mckain, M.R., Smith, R.D., Teresi, S.J., Nelson, A.D., and Wai, C.M. (2019). Origin and evolution of the octoploid strawberry genome. *Nature genetics* 51**,** 541-547.

Egawa, Y., Bujang, I., Chotechuen, S., Tomooka, N., and Tateishi, Y. (1996a). Phylogenetic differentiation of tetraploid Vigna species, V. glabrescens and V. reflexo-pilosa. *JIRCAS Journal for Scientific Papers (Japan)*.

Egawa, Y., Chotechuen, S., Tomooka, N., Thavarasook, C., and Kitbamroong, C. (1996b). *Cross-compatibility among the subgenus Ceratotropis of the genus Vigna.* Japan: Japan International Research Center for Agricultural Science.

Egawa, Y., Siriwardhane, D., Tomooka, N., Tomita, K., and Nakano, H. (1990). *Collection of V. ruikiuensis and V. reflexo-pilosa, wild relatives of adzuki bean on Iriomote island, Okinawa prefecture. .* Tsukuba, Japan: Natl. Inst. Agrobiol. Resour., National Institute of Agrobiological Resources.

Emms, D.M., and Kelly, S. (2019). OrthoFinder: phylogenetic orthology inference for comparative genomics. *Genome Biology* 20**,** 238. doi: 10.1186/s13059-019-1832-y.

Galbraith, D.W., Harkins, K.R., Maddox, J.M., Ayres, N.M., Sharma, D.P., and Firoozabady, E. (1983). Rapid Flow Cytometric Analysis of the Cell Cycle in

Intact Plant Tissues. *Science* 220, 1049-1051. doi: doi:10.1126/science.220.4601.1049.

Gordon, S.P., Levy, J.J., and Vogel, J.P. (2019). PolyCRACKER, a robust method for the unsupervised partitioning of polyploid subgenomes by signatures of repetitive DNA evolution. *BMC genomics* 20, 1-14.

Grover, C.E., Gallagher, J.P., Szadkowski, E.P., Yoo, M.J., Flagel, L.E., and Wendel, J.F. (2012). Homoeolog expression bias and expression level dominance in allopolyploids. *New Phytologist* 196, 966-971. doi: https://doi.org/10.1111/j.1469-8137.2012.04365.x.

Haas, B.J., Salzberg, S.L., Zhu, W., Pertea, M., Allen, J.E., Orvis, J., White, O., Buell, C.R., and Wortman, J.R. (2008). Automated eukaryotic gene structure annotation using EVidenceModeler and the program to assemble spliced alignments. *Genome Biol* 9, R7. doi: 10.1186/gb-2008-9-1-r7.

Hovav, R., Udall, J.A., Chaudhary, B., Rapp, R., Flagel, L., and Wendel, J.F. (2008). Partitioned expression of duplicated genes during development and evolution of a single cell in a polyploid plant. *Proceedings of the National Academy of Sciences* 105, 6191-6195.

Huang, X., Adams, M.D., Zhou, H., and Kerlavage, A.R. (1997). A tool for analyzing and annotating genomic sequences. *Genomics* 46, 37-45. doi: 10.1006/geno.1997.4984.

Jackson, S., and Chen, Z.J. (2010). Genomic and expression plasticity of polyploidy. *Current opinion in plant biology* 13, 153-159.

Javadi, F., Tun, Y.T., Kawase, M., Guan, K., and Yamaguchi, H. (2011). Molecular phylogeny of the subgenus Ceratotropis (genus Vigna, Leguminosae) reveals three eco-geographical groups and Late Pliocene–Pleistocene diversification: evidence from four plastid DNA region sequences. *Annals of Botany* 108, 367-380.

Koren, S., Walenz, B.P., Berlin, K., Miller, J.R., Bergman, N.H., and Phillippy, A.M. (2017). Canu: scalable and accurate long-read assembly via adaptive k-mer weighting and repeat separation. *Genome Res* 27, 722-736. doi: 10.1101/gr.215087.116.

Kriventseva, E.V., Tegenfeldt, F., Petty, T.J., Waterhouse, R.M., Simao, F.A., Pozdnyakov, I.A., Ioannidis, P., and Zdobnov, E.M. (2015). OrthoDB v8: update of the hierarchical catalog of orthologs and the underlying free software. *Nucleic Acids Res* 43, D250-256. doi: 10.1093/nar/gku1220.

Krzywinski, M.I., Schein, J.E., Birol, I., Connors, J., Gascoyne, R., Horsman, D., Jones, S.J., and Marra, M.A. (2009). Circos: An information aesthetic for comparative genomics. *Genome Research* 19, 1639-1645. doi: 10.1101/gr.092759.109.

Li, L., Stoeckert, C.J., Jr., and Roos, D.S. (2003). OrthoMCL: identification of ortholog groups for eukaryotic genomes. *Genome Res* 13, 2178-2189. doi: 10.1101/gr.1224503.

Lieberman-Aiden, E., Van Berkum, N.L., Williams, L., Imakaev, M., Ragoczy, T., Telling, A., Amit, I., Lajoie, B.R., Sabo, P.J., Dorschner, M.O., Sandstrom, R., Bernstein, B., Bender, M.A., Groudine, M., Gnirke, A., Stamatoyannopoulos, J., Mirny, L.A., Lander, E.S., and Dekker, J. (2009). Comprehensive mapping of long-range interactions reveals folding principles of the human genome. *Science* 326, 289-293. doi: 10.1126/science.1181369.

Maréchal, R. (1978). Etude taxonomique d'un groupe complexe d'espèces des genres Phaseolus et Vigna (Papilionaceae) sur la base de données morphologiques et polliniques, traitées par l'analyse informatique. *Boissiera* 28**,** 1-273.
Nettleton, D., Flagel, L., Udall, J., and Wendel, J. (2008). Duplicate Gene Expression in Allopolyploid Gossypium Reveals Two Temporally Distinct Phases of Expression Evolution.
Norihiko, T., Kaga, A., Isemura, T., Vaughan, D., Srinives, P., Somta, P., Thadavong, S., Bounphanousay, C., Kanyavong, K., and Inthapanya, P. (Year). "Vigna genetic resources", in: *Proceeding of the14th NIAS international workshop on Genetic Resources, Genetic and Comparative Genomics of Legumes (Glycine and Vigna)*), 11-21.
Pootakham, W., Naktang, C., Sonthirod, C., Kongkachana, W., Narong, N., Sangsrakru, D., Maknual, C., Jiumjamrassil, D., Chumriang, P., and Tangphatsornroung, S. (2022). Chromosome-level genome assembly of the Indian mangrove (*Ceriops tagal*) revealed a genome-wide duplication event predating the divergence of Rhizophoraceae mangroves. *Plant Genome* 00**,** e20217. doi: doi.org/10.1002/tpg2.20217.
Powell, J., Fitzgerald, T., Stiller, J., Berkman, P., Gardiner, D., Manners, J., Henry, R., and Kazan, K. (2016). The defence-associated transcriptome of hexaploid wheat displays homoeolog expression and induction bias. *Plant Biotechnology Journal* 15. doi: 10.1111/pbi.12651.
Price, A.L., Jones, N.C., and Pevzner, P.A. (2005). De novo identification of repeat families in large genomes. *Bioinformatics* 21**,** i351-i358.
Putnam, N.H., O'connell, B.L., Stites, J.C., Rice, B.J., Blanchette, M., Calef, R., Troll, C.J., Fields, A., Hartley, P.D., Sugnet, C.W., Haussler, D., Rokhsar, D.S., and Green, R.E. (2016). Chromosome-scale shotgun assembly using an in vitro method for long-range linkage. *Genome Res* 26**,** 342-350. doi: 10.1101/gr.193474.115.
Rapp, R.A., Udall, J.A., and Wendel, J.F. (2009). Genomic expression dominance in allopolyploids. *BMC Biology* 7**,** 18. doi: 10.1186/1741-7007-7-18.
Renny-Byfield, S., Rodgers-Melnick, E., and Ross-Ibarra, J. (2017). Gene fractionation and function in the ancient subgenomes of maize. *Molecular biology and evolution* 34**,** 1825-1832.
Schultz, M.D., Schmitz, R.J., and Ecker, J.R. (2012). 'Leveling'the playing field for analyses of single-base resolution DNA methylomes. *Trends in Genetics* 28**,** 583-585.
Sebastian, P., Schaefer, H., Telford, I.R., and Renner, S.S. (2010). Cucumber (Cucumis sativus) and melon (C. melo) have numerous wild relatives in Asia and Australia, and the sister species of melon is from Australia. *Proceedings of the National Academy of Sciences* 107**,** 14269-14273.
Simão, F.A., Waterhouse, R.M., Ioannidis, P., Kriventseva, E.V., and Zdobnov, E.M. (2015). BUSCO: assessing genome assembly and annotation completeness with single-copy orthologs. *Bioinformatics* 31**,** 3210-3212. doi: 10.1093/bioinformatics/btv351.
Stamatakis, A. (2006). RAxML-VI-HPC: maximum likelihood-based phylogenetic analyses with thousands of taxa and mixed models. *Bioinformatics* 22**,** 2688-2690. doi: btl446 [pii]

10.1093/bioinformatics/btl446.
Stanke, M., Steinkamp, R., Waack, S., and Morgenstern, B. (2004). AUGUSTUS: a web server for gene finding in eukaryotes. *Nucleic Acids Research* 32**,** W309-W312. doi: 10.1093/nar/gkh379.
Swindell, R.E., Watt, E.E., and Evans, G.M. (1973). "A natural tetraploid mungbean of suspected amphidiploid origin". Oxford University Press).
Tateishi, Y. (1985). A revision of the azuki bean group, the subgenus Ceratotropis of the genus Vigna (Leguminosae. *Ph. D. Thesis, Tohoku University*.
Tateishi, Y., and Ohashi, H. (1990). "Systematics of the azuki bean group in the genus Vigna," in *Bruchids and legumes: economics, ecology and coevolution*. Springer), 189-199.
Thomas, B.C., Pedersen, B., and Freeling, M. (2006). Following tetraploidy in an Arabidopsis ancestor, genes were removed preferentially from one homeolog leaving clusters enriched in dose-sensitive genes. *Genome research* 16**,** 934-946.
Tomooka, N., Vaughan, D.A., Moss, H., and Maxted, N. (2002). *The Asian Vigna: Genus Vigna Subgenus Ceratotropis Genetic Resources.*: Kluwer, Dordrecht.
Tun, Y.T., and Yamaguchi, H. (2007). Phylogenetic relationship of wild and cultivated Vigna (Subgenus Ceratotropis, Fabaceae) from Myanmar based on sequence variations in non-coding regions of trnT-F. *Breeding Science* 57**,** 271-280.
Wang, Y., Tang, H., Debarry, J.D., Tan, X., Li, J., Wang, X., Lee, T.H., Jin, H., Marler, B., Guo, H., Kissinger, J.C., and Paterson, A.H. (2012). MCScanX: a toolkit for detection and evolutionary analysis of gene synteny and collinearity. *Nucleic Acids Res* 40**,** e49. doi: 10.1093/nar/gkr1293.
Woodhouse, M.R., Cheng, F., Pires, J.C., Lisch, D., Freeling, M., and Wang, X. (2014). Origin, inheritance, and gene regulatory consequences of genome dominance in polyploids. *Proceedings of the National Academy of Sciences* 111**,** 5283-5288.
Wu, J., Lin, L., Xu, M., Chen, P., Lui, D., Sun, Q., Ran, L., and Wang, Y. (2018). Homoeolog expression bias and expression level dominance in resynthesized allopolyploid *Brassica napus*. *BMC Genomics* 19**,** 586.
Wu, T., and Watanabe, C. (2005). GMAP: a genomic mapping and alignment program for mRNA and EST sequences. *Bioinformatics* 21**,** 1859 - 1875.
Yang, Z. (2007). PAML 4: phylogenetic analysis by maximum likelihood. *Mol Biol Evol* 24**,** 1586-1591. doi: 10.1093/molbev/msm088.
Yoo, M.-J., Szadkowski, E., and Wendel, J. (2012). Homoeolog expression bias and expression level dominance in allopolyploid cotton. *Heredity* 110. doi: 10.1038/hdy.2012.94.
Zhou, Q., Lim, J.-Q., Sung, W.-K., and Li, G. (2019). An integrated package for bisulfite DNA methylation data analysis with Indel-sensitive mapping. *BMC bioinformatics* 20**,** 1-11.

Figure 1

Click here to access/download;Figure;Figure 1 - CIRCOS.jpeg

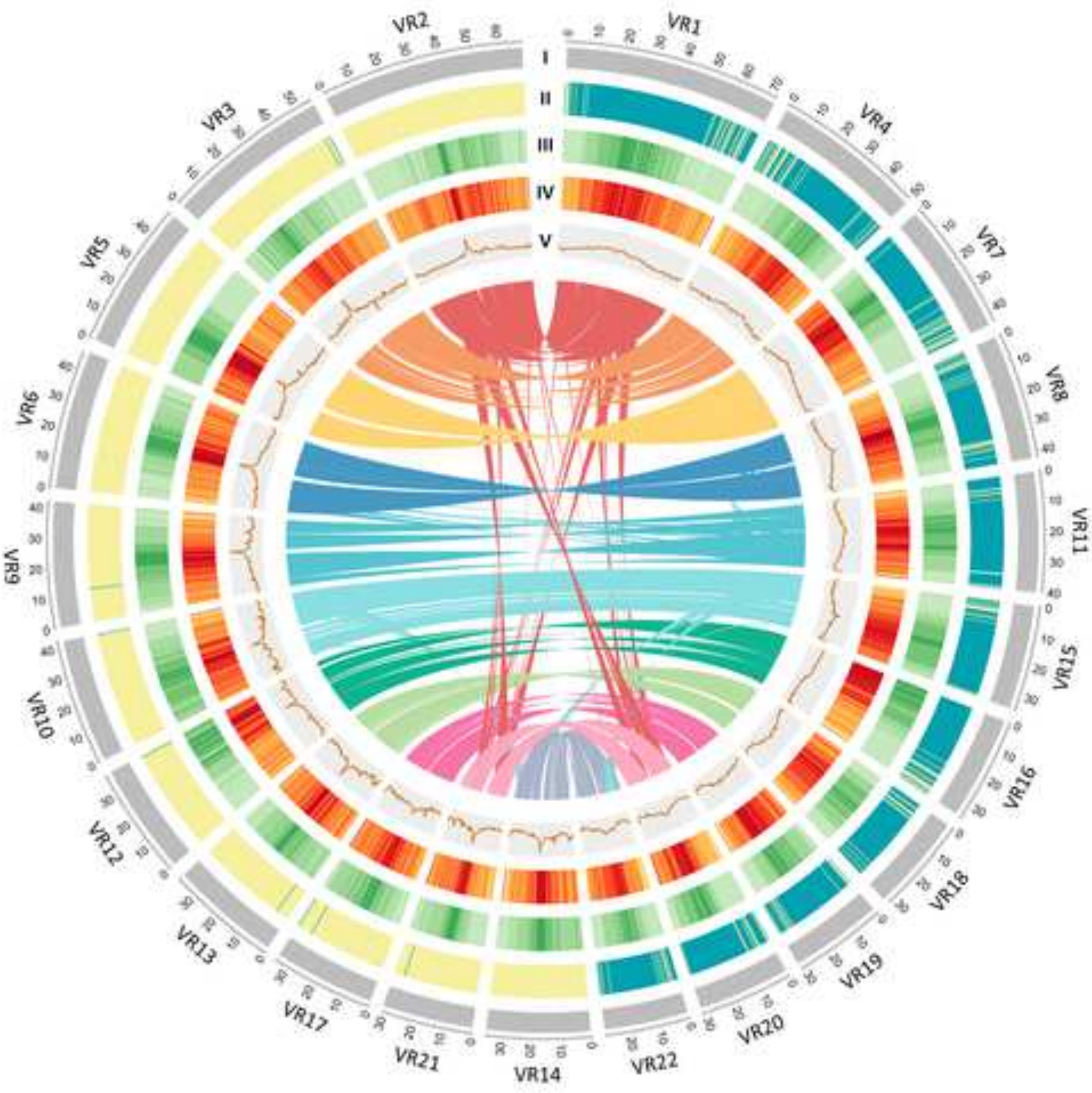

Figure 2 Click here to access/download;Figure;Figure 2 - phylogeny 4DTv.jpeg

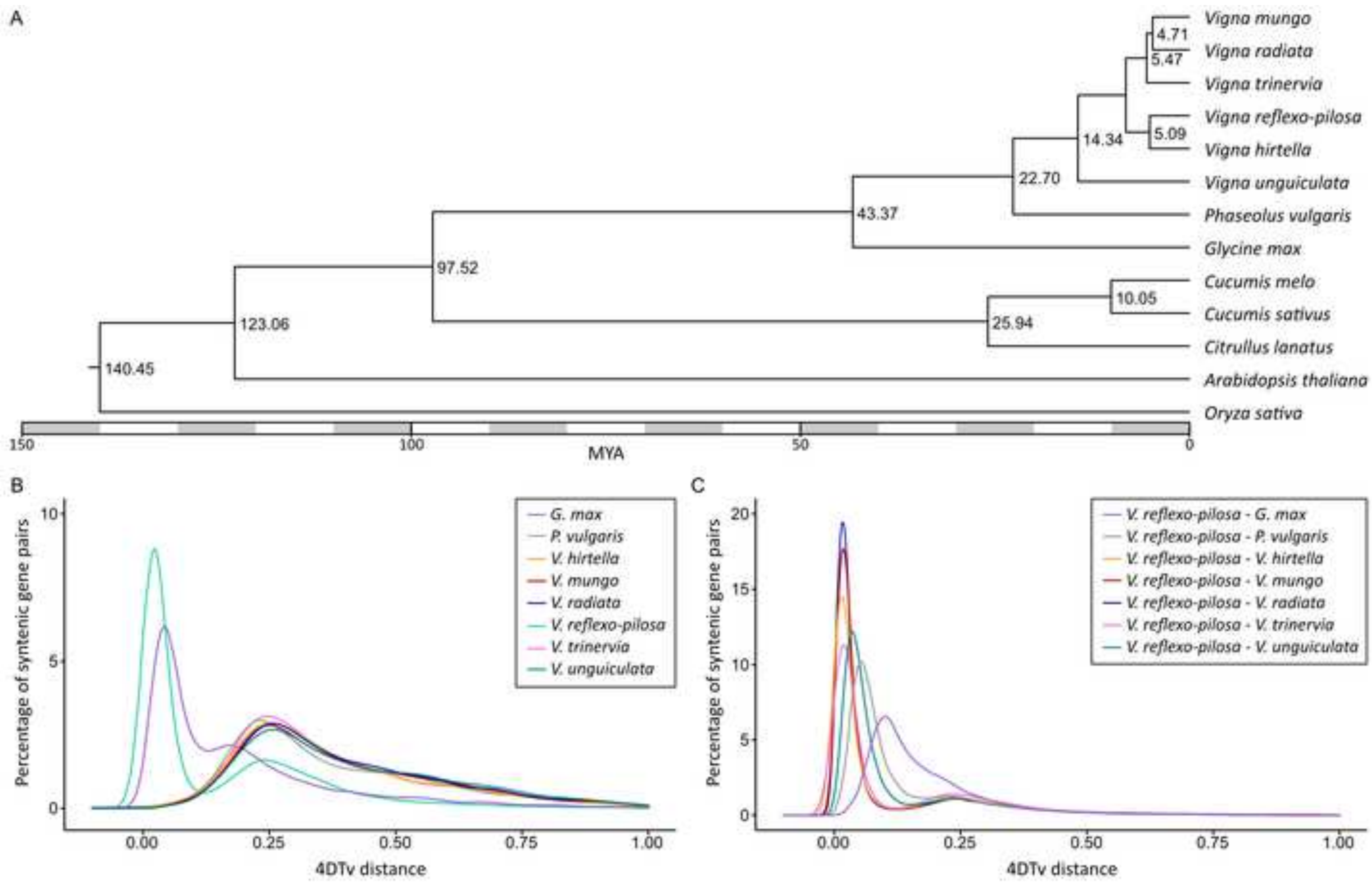

Figure 3

Click here to access/download;Figure;Figure 3 - Homoeolog expression bias.jpeg

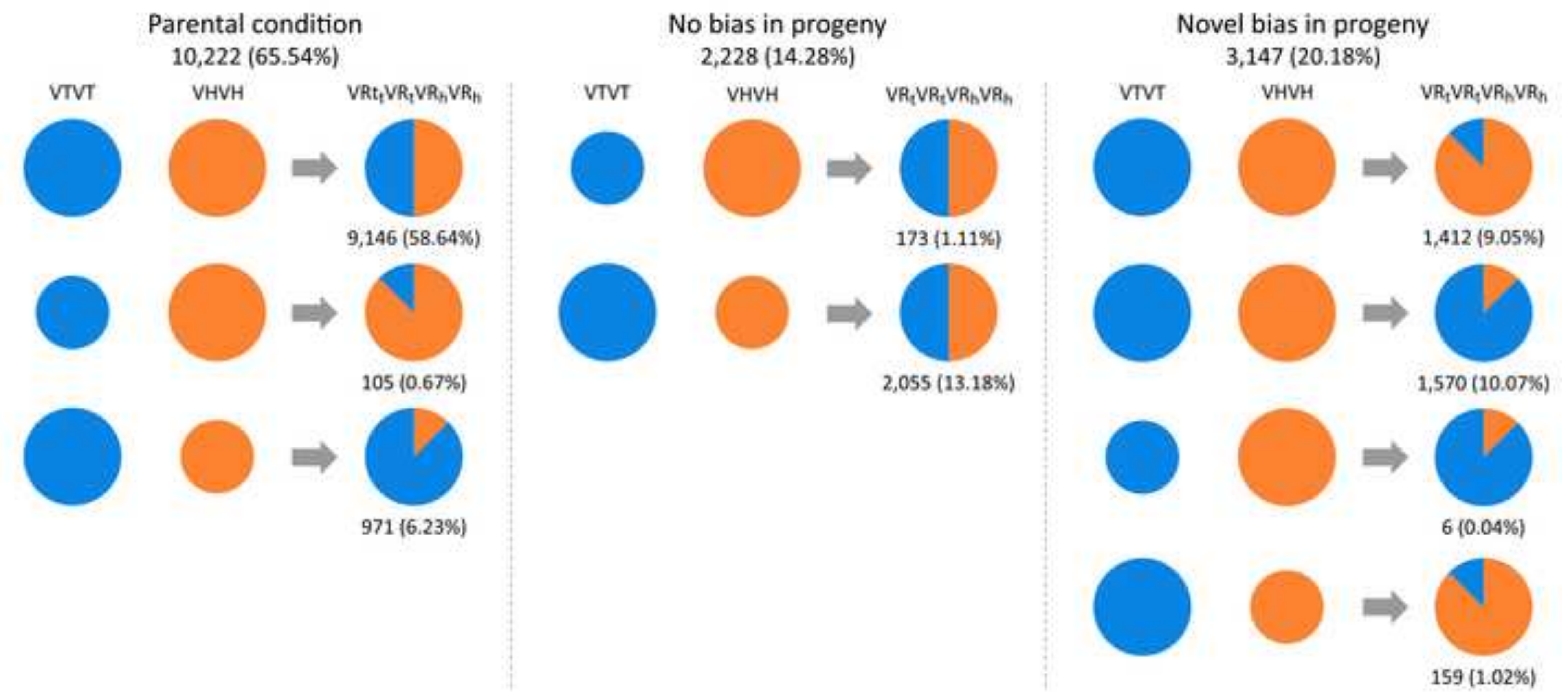

Figure 4

Click here to access/download;Figure;Figure 4 - ELD.jpeg

| Additivity | | ELD-VH | | ELD-VT | | Transgressive down-regulation | | | Transgressive up-regulation | | | No Change | Total |
|---|---|---|---|---|---|---|---|---|---|---|---|---|---|
| I | XII | II | XI | IV | IX | III | VII | X | V | VI | VIII | | |
| VT VR VH | VT VR VH | VT VR VH | VT VR VH | VT VR VH | VT VR VH | VT VR VH | VT VR VH | VT VR VH | VT VR VH | VT VR VH | VT VR VH | | |
| 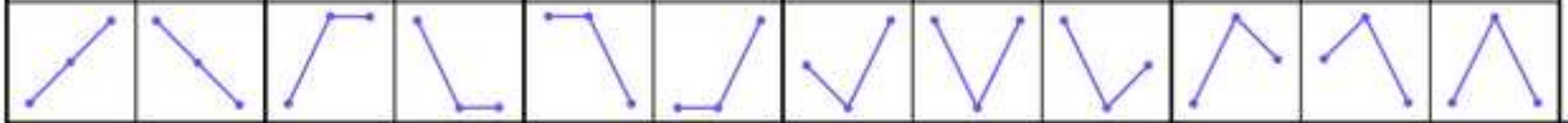 | | | | | | | | | | | | | |
| 54 | 0 | 352 | 77 | 5,205 | 18 | 0 | 5 | 0 | 145 | 1,178 | 5,105 | 3,458 | 15,597 |
| 0.35% | 0% | 2.26% | 0.49% | 33.37% | 0.12% | 0% | 0.03% | 0% | 0.93% | 7.55% | 32.73% | 22.17% | |

Figure 5 Click here to access/download;Figure;Figure 5 - Methylation Vr Vh Vt.jpeg

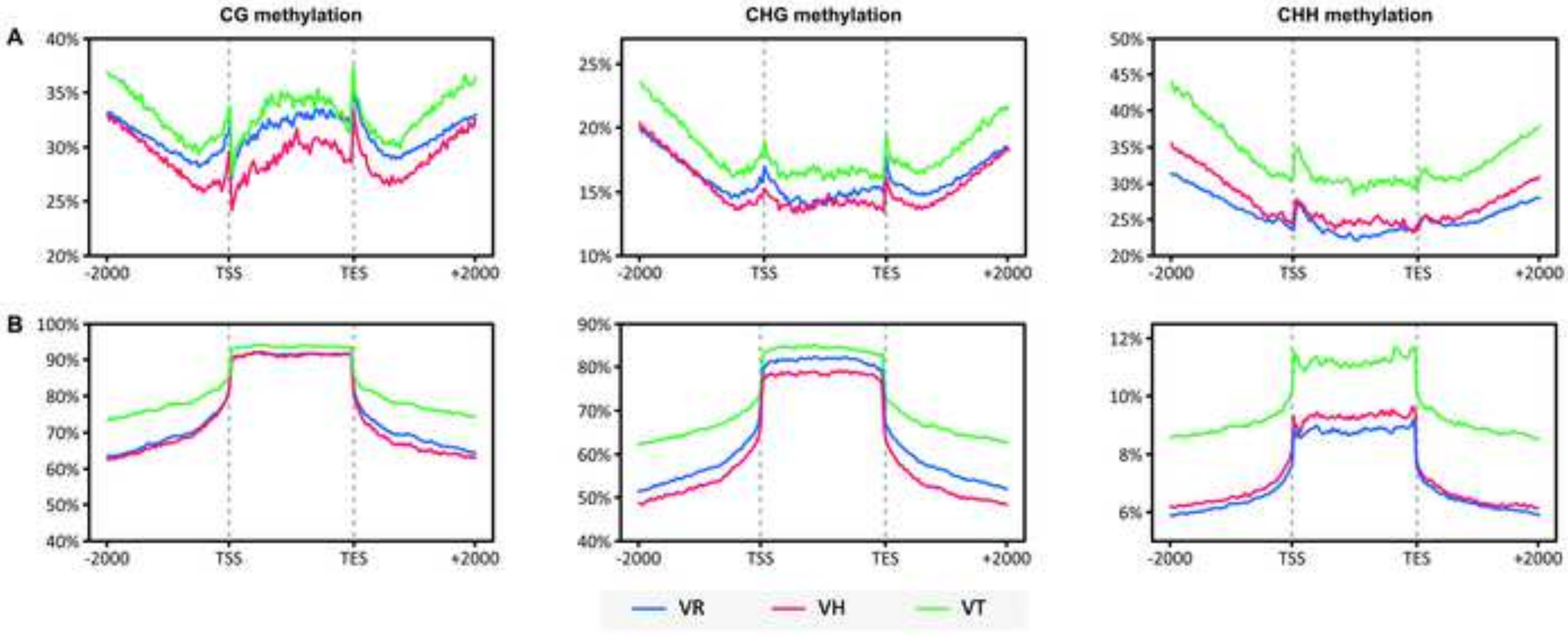

Figure 6

Click here to access/download;Figure;Figure 6 - subgenome methylation.jpeg

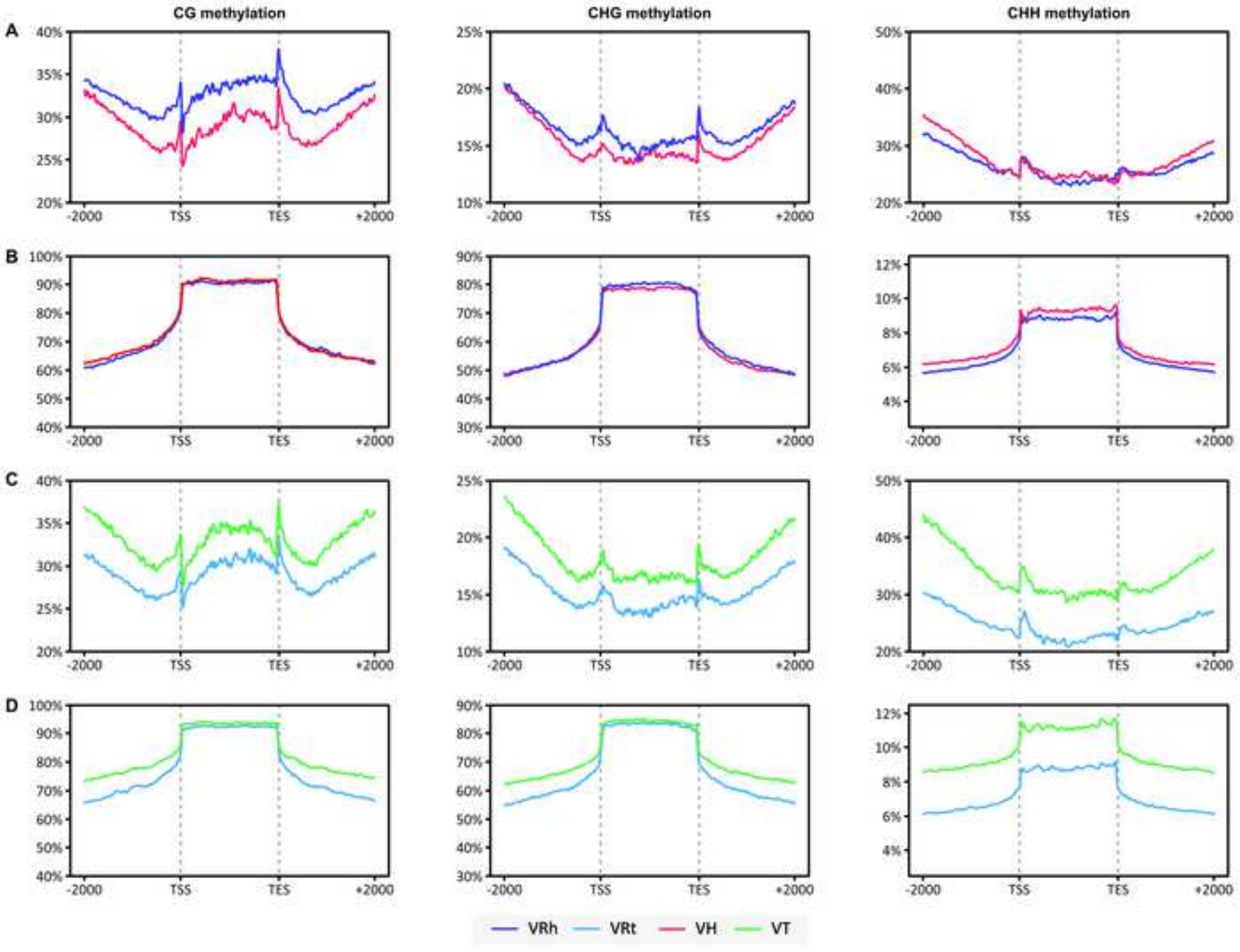

Click here to access/download
**Supplementary Material**
Supplementary figures.pdf
