# Supplementary material for: Genome assemblies of Vigna reflexo-pilosa (créole bean) and its progenitors, Vigna hirtella and Vigna trinervia, revealed homoeolog expression bias and expression-level dominance in the allotetraploid
Source: Gigascience. 2023 Jul 20;12:giad050. doi: 10.1093/gigascience/giad050 (PMC10357499; doi:10.1093/gigascience/giad050)
Supplement: giad050_Supplemental_Figures_and_Tables [file giad050_supplemental_figures_and_tables.zip › Supplementary figures.pdf]

Genome assemblies of *Vigna reflex-pilosa* (créole bean) and its progenitors, *Vigna hirtella* and *Vigna trinervia*, revealed homoeolog expression bias and expression level dominance in the allotetraploid

Wirulda Pootakham[1,*], Chutima Sonthirod[1,†], Chaiwat Naktang[1,†], Chutintorn Yundaeng[1], Thippawan Yoocha[1], Wasitthee Kongkachana[1], Duangjai Sangsrakru[1], Prakit Somta[2], Sithichoke Tangphatsornruang[1]

[1]National Omics Center, National Science and Technology Development Agency (NSTDA), Pathum Thani, Thailand.
[2]Department of Agronomy, Faculty of Agriculture at Kamphaeng Saen, Kasetsart University, Nakhon Pathom, Thailand.

[†]Equal contribution

*Corresponding authors
Wirulda Pootakham (wirulda@alumni.stanford.edu)

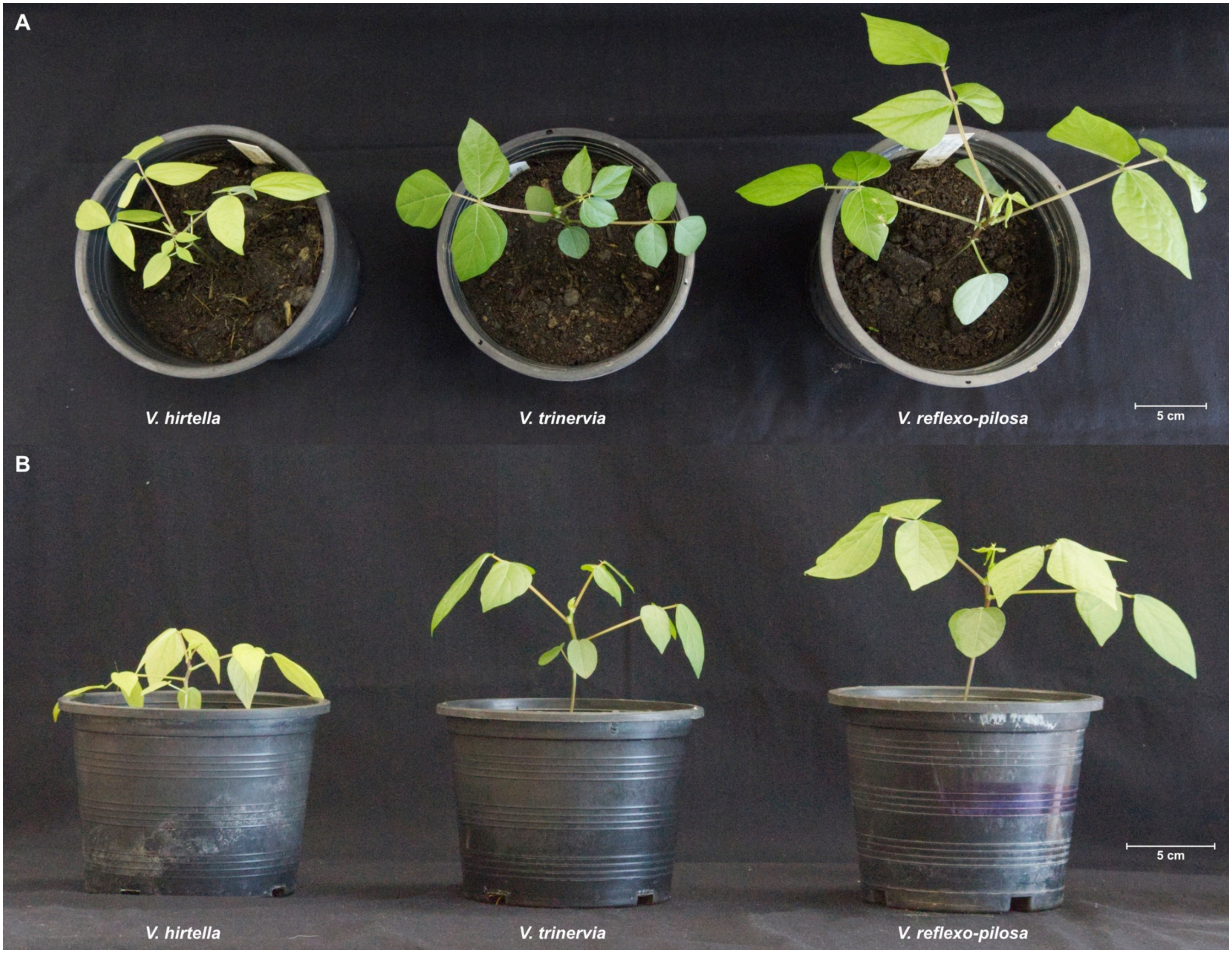

Supplementary Figure 1. (A) A top and **(B)** side view of three-week-old *V. reflexo-pilosa* and its two progenitors, *V. hirtella* and *V. trinervia*.

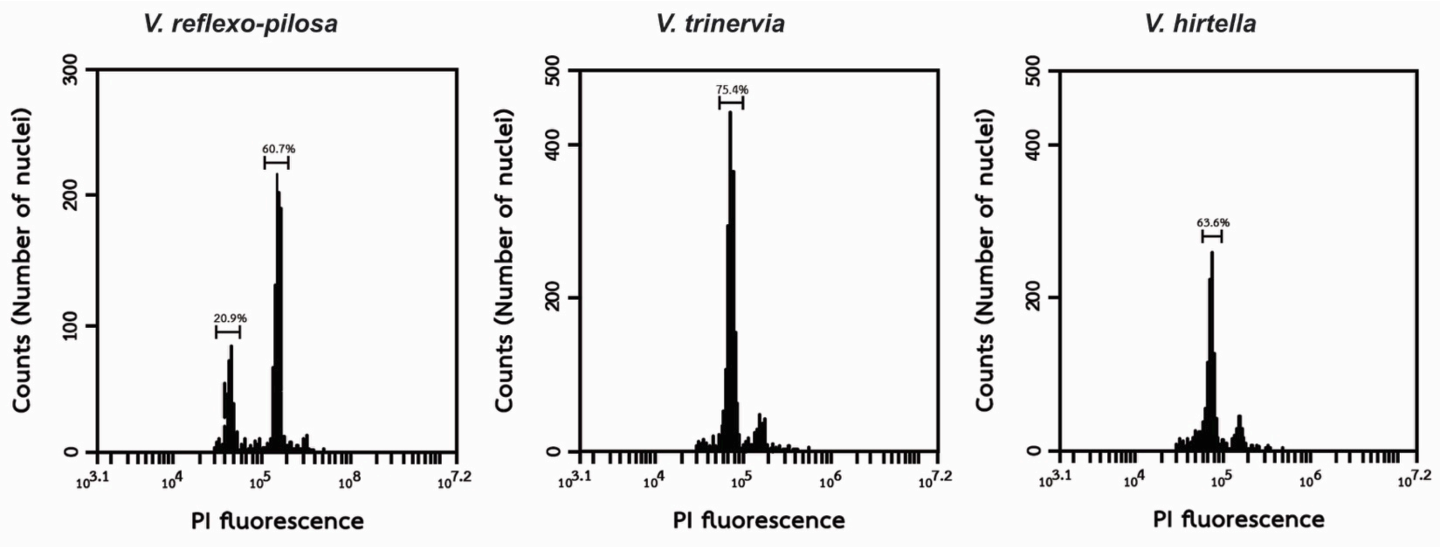

Supplementary Figure S2. Genome size estimation by DNA flow cytometry.

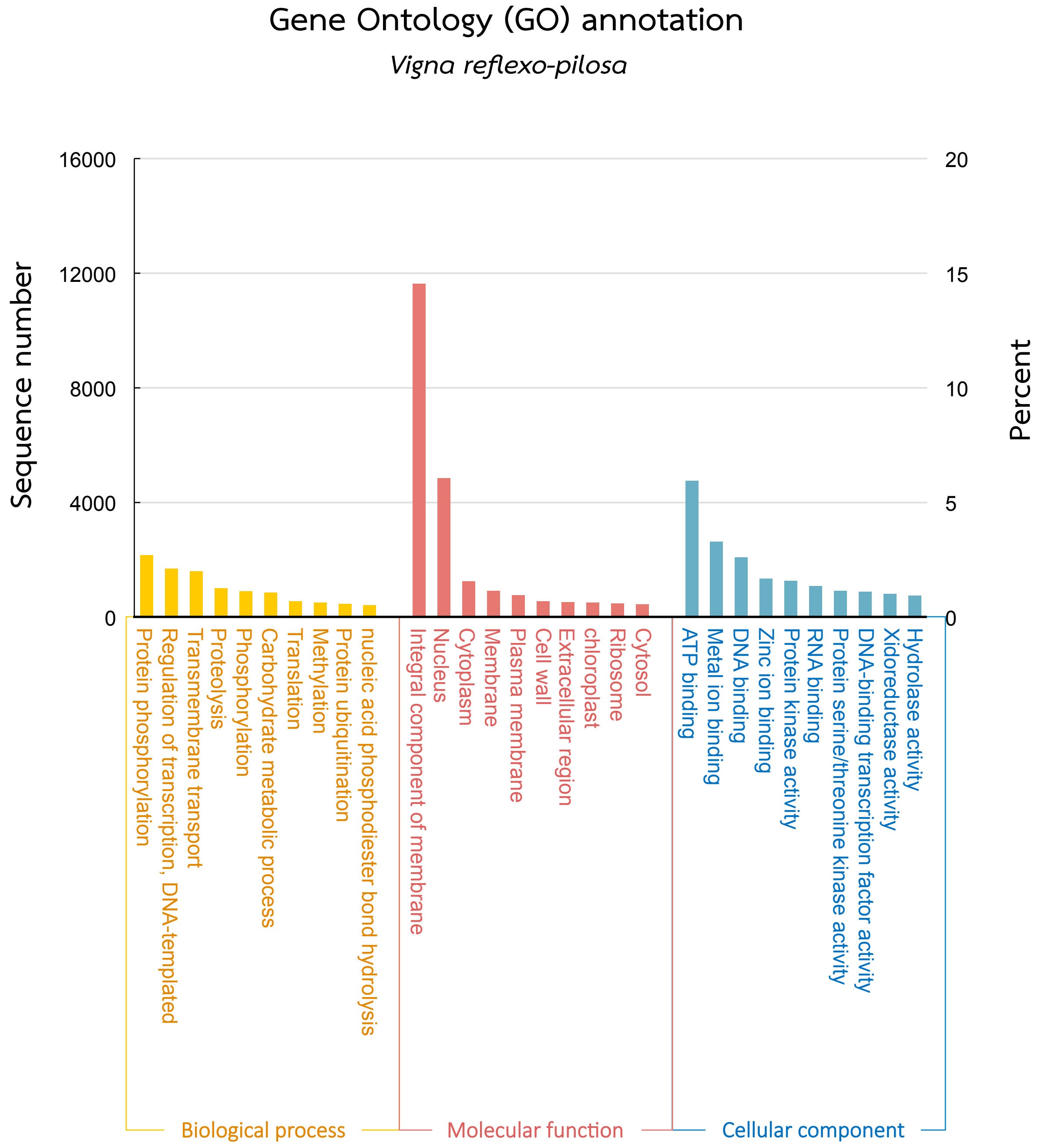

Supplementary Figure 3. GO annotation statistics for *V. reflexo-pilosa.*

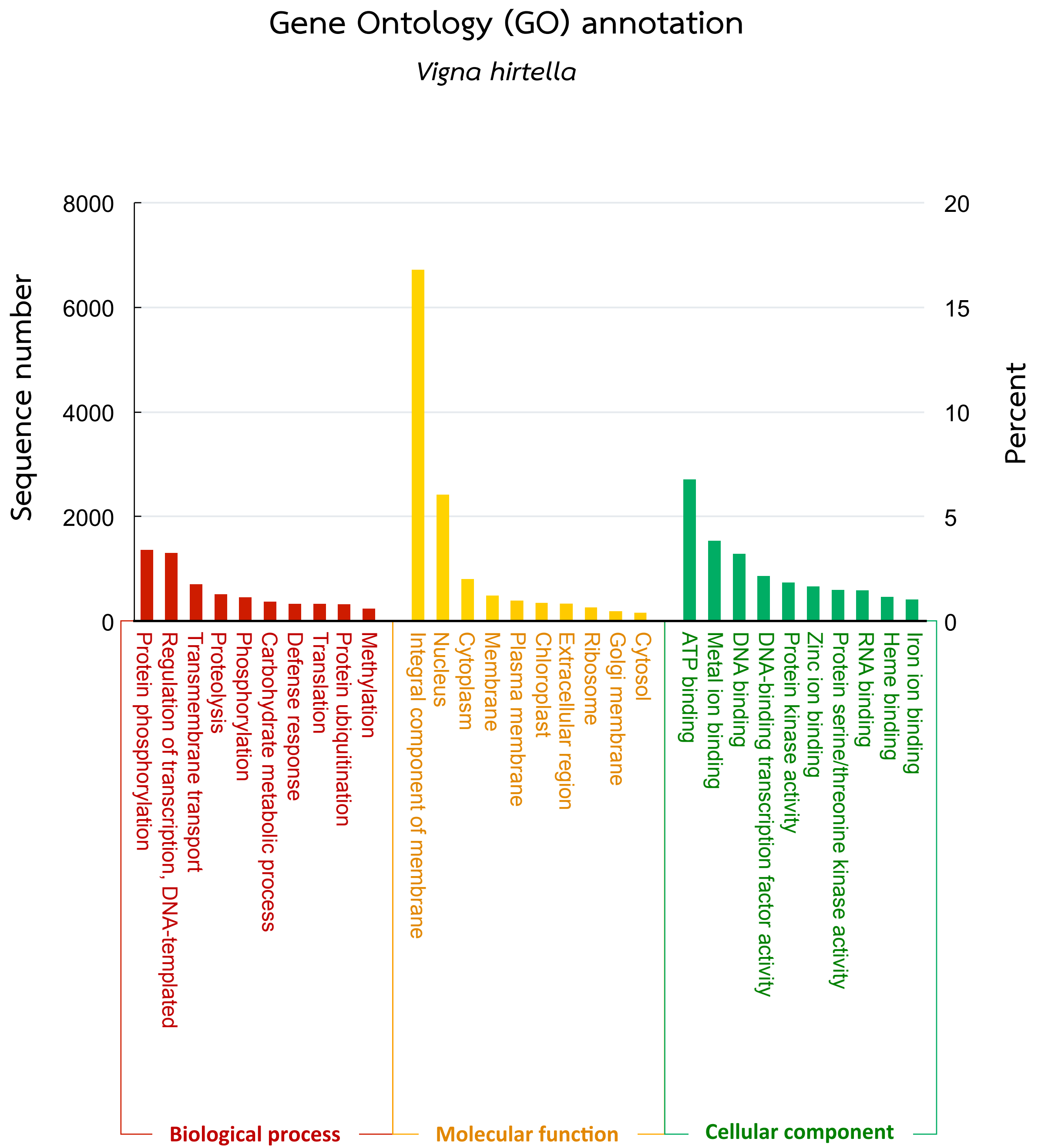

Supplementary Figure 4. GO annotation statistics for *V. hirtella.*

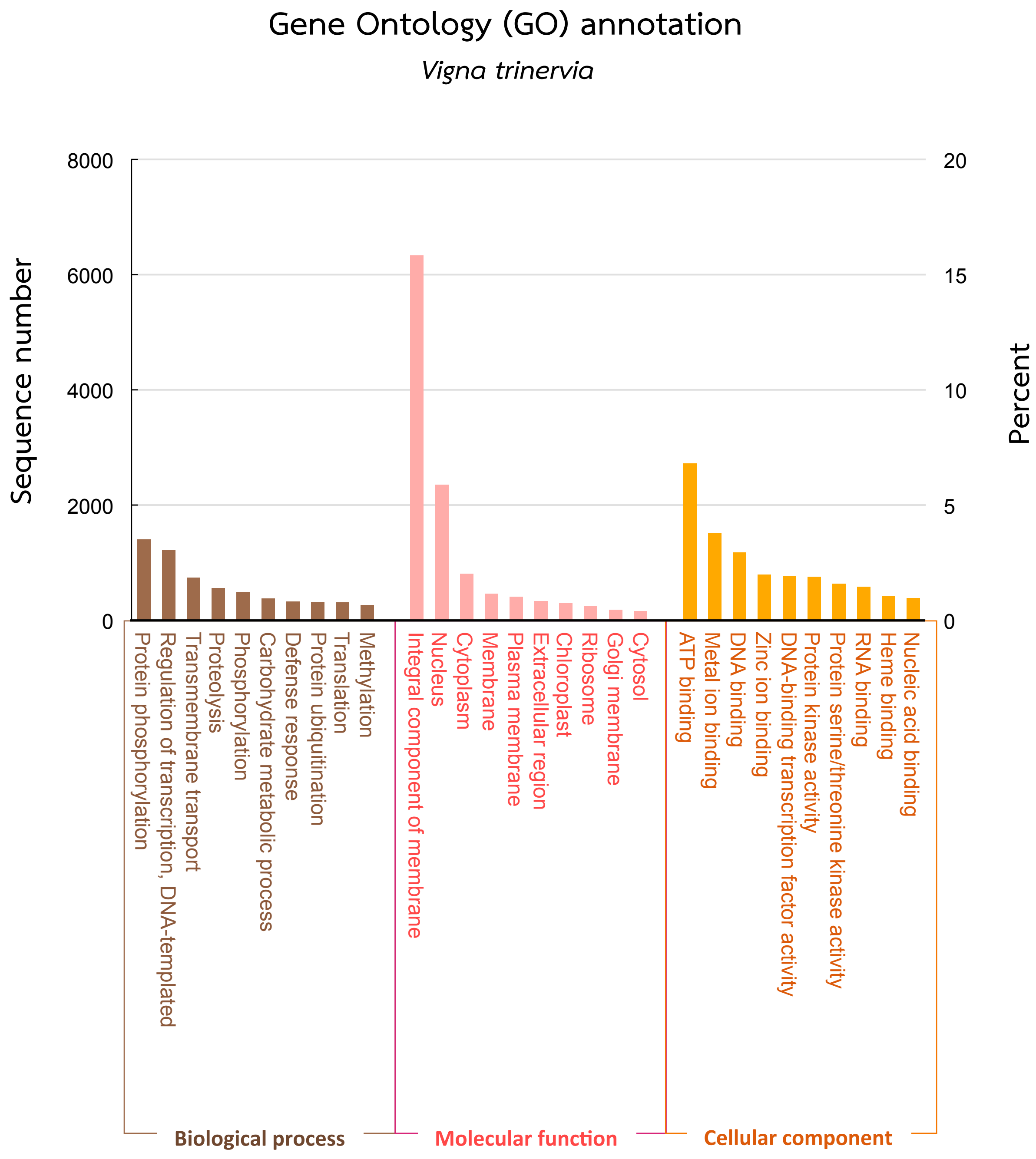

Supplementary Figure 5. GO annotation statistics for *V. trinervia.*

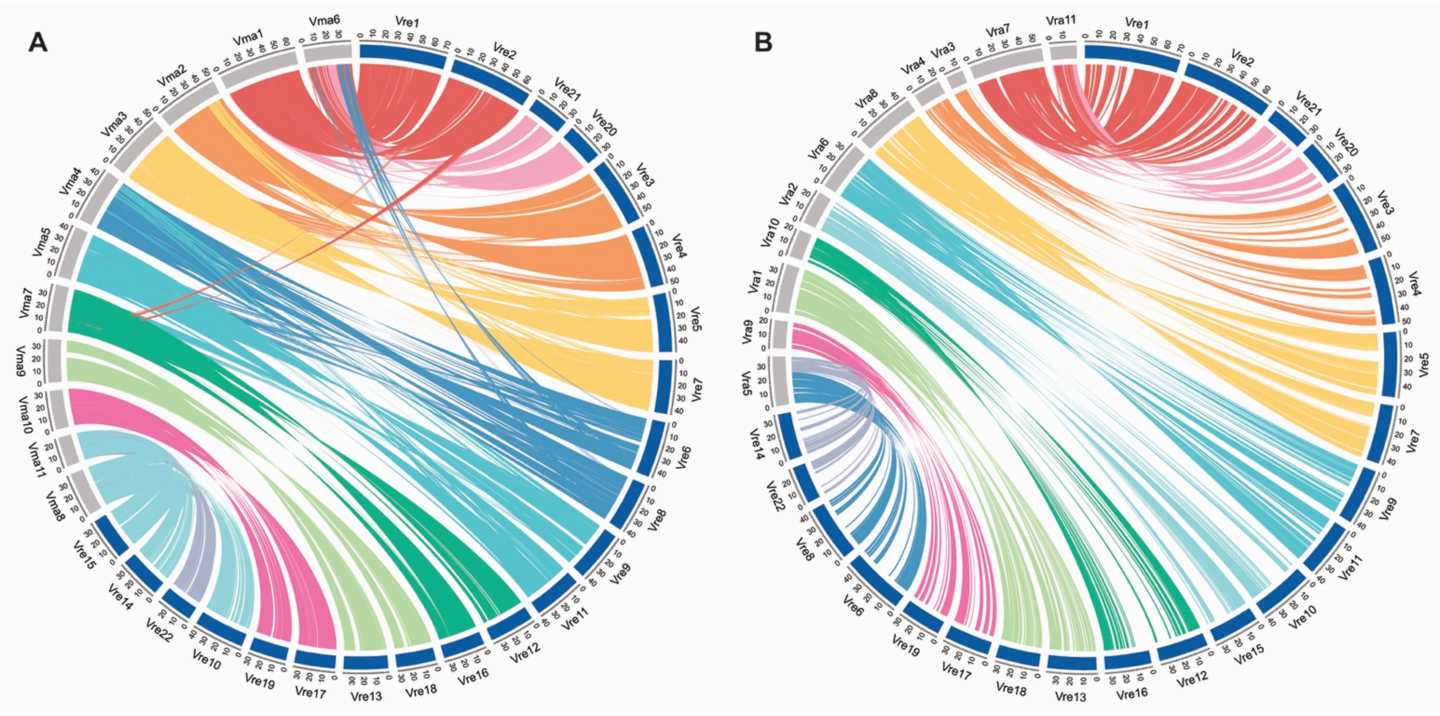

Supplementary Figure 6. Synteny between *V. reflexo-pilosa* and *V. mungo* (A) and *V. radiata* (B).
