# Supplementary material for: Genome assemblies of Vigna reflexo-pilosa (créole bean) and its progenitors, Vigna hirtella and Vigna trinervia, revealed homoeolog expression bias and expression-level dominance in the allotetraploid
Source: Gigascience. 2023 Jul 20;12:giad050. doi: 10.1093/gigascience/giad050 (PMC10357499; doi:10.1093/gigascience/giad050)
Supplement: giad050_Response_to_Reviewer_Comments_Original_Submission [file giad050_response_to_reviewer_comments_original_submission.pdf]

Author's Response To Reviewer Comments

Close

Reviewers’ comments
Reviewer 1:
The authors generated chromosomal level genome assembly for three Vigna bean genomes, including the tetraploid V. reflex-pilosa via long read sequencing and two other diploid genomes with short sequencing. The genome assembly and associated data such as annotation is useful for the genetic and genomic research of this field. In addition, the authors compared the homoeolog gene expression with its diploid progenitors, and found the bias and expression level dominance. There are few comments, as detailed below.

1. In Section DNA and RNA sequencing library preparation at line 118, which type of PacBio long reads were generated, CLR, CCS or HiFi, which is not clear?

RESPONSE: We added the detail (Line 126) indicating that the CCS reads were generated from the long-read PacBio platform.

2. The estimated genome size was generated via flow cytometry, which should be a range of estimated size, not an exact size, like 900 M.

RESPONSE: We provided the estimated genome sizes in ranges along with their respective average sizes (for the purpose of calculating the genome coverage) obtained from the flow cytometry.

3. What is observed for the gene amplification/loss after becoming tetraploid compared with the two diploid progenitors? The authors compared the difference at expression level but not at the genomic DNA level, which is present in the genome assembly. It is better to discussion or add the difference in the main context.

RESPONSE: We analyzed the gene expansion/contraction in the tetraploid vs its two progenitors (Line 388) and showed the results in Supplementary Table S1/S2.

Reviewer 2:
Comments This is a comprehensive and well-designed study, with some minor issues with writing, such as missing citations for bioinformatics tools like Supernova, BWA, HiRise, minimap2, etc., and missing version information for tools like MUSCLE alignment.

Supernova Weisenfeld, N. I., Kumar, V., Shah, P., Church, D. M., & Jaffe, D. M. (2017). Direct determination of diploid genome sequences. Genome Research, 27(5), 757-767. https://doi.org/10.1101/gr.214874.116

BWA Li, H., & Durbin, R. (2009). Fast and accurate short read alignment with Burrows-Wheeler transform. Bioinformatics, 25(14), 1754-1760. https://doi.org/10.1093/bioinformatics/btp324

HiRise Putnam, N. H., O'Connell, B., Stites, J. C., Rice, B. J., Blanchette, M., Calef, R., Troll, C. J., Fields, A. T., Hartley, P. S., Sugnet, C. W., Haussler, D., Rokhsar, D. S., & Green, R. (2016). Chromosome-scale shotgun assembly using an in vitro method for long-range linkage. Genome Research, 26(3), 342-350. https://doi.org/10.1101/gr.193474.115

Minmap2 Li, H. (2018). Minimap2: pairwise alignment for nucleotide sequences. Bioinformatics, 34(18), 3094-3100. https://doi.org/10.1093/bioinformatics/bty191

RepeatModeler2 Flynn, J. M., Hubley, R., Goubert, C., Rosen, J., Clark, A. G., Feschotte, C., & Smit, A. F. (2020). RepeatModeler2 for automated genomic discovery of transposable element families. Proceedings of the National Academy of Sciences of the United States of America, 117(17), 9451-9457. https://doi.org/10.1073/pnas.1921046117

RESPONSE: References to bioinformatic tools and the version of MUSCLE have been added to the manuscript as suggested.

Close
