# Supplementary material for: Genome assemblies of Vigna reflexo-pilosa (créole bean) and its progenitors, Vigna hirtella and Vigna trinervia, revealed homoeolog expression bias and expression-level dominance in the allotetraploid
Source: Gigascience. 2023 Jul 20;12:giad050. doi: 10.1093/gigascience/giad050 (PMC10357499; doi:10.1093/gigascience/giad050)
Supplement: giad050_Response_to_Reviewer_Comments_Revision_1 [file giad050_response_to_reviewer_comments_revision_1.pdf]

Author's Response To Reviewer Comments

Close

We have added the references the database DOI as requested.

Close
