# Supplementary material for: Genome assemblies of Vigna reflexo-pilosa (créole bean) and its progenitors, Vigna hirtella and Vigna trinervia, revealed homoeolog expression bias and expression-level dominance in the allotetraploid
Source: Gigascience. 2023 Jul 20;12:giad050. doi: 10.1093/gigascience/giad050 (PMC10357499; doi:10.1093/gigascience/giad050)
Supplement: giad050_Reviewer_1_Report_Original_Submission — Xuewen WANG, Ph.D -- 4/15/2023 Reviewed [file giad050_reviewer_1_report_original_submission.pdf]

**Reviewer Report**

**Title: Genome assemblies of Vigna reflexo-pilosa (crÃ©ole bean) and its progenitors, Vigna hirtella and Vigna trinervia, revealed homoeolog expression bias and expression level dominance in the allotetraploid**

**Version: Original Submission** **Date:** 4/15/2023

**Reviewer name: Xuewen WANG, Ph.D.**

**Reviewer Comments to Author:**

The authors generated chromosomal level genome assembly for three Vigna bean genomes, including the tetraploid V. reflex-pilosa via long read sequencing and two other diploid genomes with short sequencing. The genome assembly and associated data such as annotation is useful for the genetic and genomic research of this field. In addition, the authors compared the homoeolog gene expression with its diploid progenitors, and found the bias and expression level dominance. There are few comments, as detailed below.1. In Section DNA and RNA sequencing library preparation at line 118, which type of PacBio long reads were generated, CLR, CCS or HiFi, which is not clear?2. The estimated genome size was generated via flow cytometry, which should be a range of estimated size, not an exact size, like 900 M.3. What is observed for the gene amplification/loss after becoming tetraploid compared with the two diploid progenitors? The authors compared the difference at expression level but not at the genomic DNA level, which is present in the genome assembly. It is better to discussion or add the difference in the main context.

**Level of Interest**

Please indicate how interesting you found the manuscript: Choose an item.

**Quality of Written English**

Please indicate the quality of language in the manuscript: Choose an item.

**Declaration of Competing Interests**

Please complete a declaration of competing interests, considering the following questions:

- Have you in the past five years received reimbursements, fees, funding, or salary from an organisation that may in any way gain or lose financially from the publication of this manuscript, either now or in the future?
- Do you hold any stocks or shares in an organisation that may in any way gain or lose financially from the publication of this manuscript, either now or in the future?
- Do you hold or are you currently applying for any patents relating to the content of the manuscript?

- Have you received reimbursements, fees, funding, or salary from an organization that holds or has applied for patents relating to the content of the manuscript?
- Do you have any other financial competing interests?
- Do you have any non-financial competing interests in relation to this paper?

If you can answer no to all of the above, write 'I declare that I have no competing interests' below. If your reply is yes to any, please give details below.

I declare that I have no competing interests.

I agree to the open peer review policy of the journal. I understand that my name will be included on my report to the authors and, if the manuscript is accepted for publication, my named report including any attachments I upload will be posted on the website along with the authors' responses. I agree for my report to be made available under an Open Access Creative Commons CC-BY license (http://creativecommons.org/licenses/by/4.0/). I understand that any comments which I do not wish to be included in my named report can be included as confidential comments to the editors, which will not be published.

Choose an item.

To further support our reviewers, we have joined with Publons, where you can gain additional credit to further highlight your hard work (see: https://publons.com/journal/530/gigascience). On publication of this paper, your review will be automatically added to Publons, you can then choose whether or not to claim your Publons credit. I understand this statement.

Yes Choose an item.
