# Supplementary material for: Genome assemblies of Vigna reflexo-pilosa (créole bean) and its progenitors, Vigna hirtella and Vigna trinervia, revealed homoeolog expression bias and expression-level dominance in the allotetraploid
Source: Gigascience. 2023 Jul 20;12:giad050. doi: 10.1093/gigascience/giad050 (PMC10357499; doi:10.1093/gigascience/giad050)
Supplement: giad050_Reviewer_2_Report_Original_Submission — Christopher Cullis -- 4/26/2023 Reviewed [file giad050_reviewer_2_report_original_submission.pdf]

Reviewer Report

Title: Genome assemblies of Vigna reflexo-pilosa (crÃ©ole bean) and its progenitors, Vigna hirtella and Vigna trinervia, revealed homoeolog expression bias and expression level dominance in the allotetraploid

Version: Original Submission **Date:** 4/26/2023

Reviewer name: Christopher Cullis

Reviewer Comments to Author:

Manuscript: Genome Assemblies of Vigna reflexo-pilosa (CrÃ©ole Bean) and Its Progenitors, Vignahirtella and Vigna trinervia, Revealed Homoeolog Expression Bias and Expression LevelDominance in the AllotetraploidSummaryThis study utilized a combination of sequencing platforms PacBio and Hi-C to produce the first high-quality chromosome-level assembly of V. reflexo-pilosa, a neglected allotetraploid Vigna species with a chromosome count of 2n=4x=44. Additionally, by using 10x Genomics and Illumina short reads, this research generated genome assemblies for its progenitor species, V. hirtellaand V. trinervia, and explore their phylogenetic relationships with other species. This study also analyzed the transcriptome data to investigate homoeolog expression bias, expression level dominance, and changes in DNA methylation pattern following the hybridization and polyploidization during the formation of V. reflexo-pilosa, providing insights for the breeding of Vignaspecies.CommentsThis is a comprehensive and well-designed study, with some minor issues with writing, such as missing citations for bioinformatics tools like Supernova, BWA, HiRise, minimap2, etc., and missing version information for tools like MUSCLE alignment.SupernovaWeisenfeld, N. I., Kumar, V., Shah, P., Church, D. M., & Jaffe, D. M. (2017). Direct determination of diploid genome sequences. Genome Research, 27(5), 757-767. https://doi.org/10.1101/gr.214874.116BWALi, H., & Durbin, R. (2009). Fast and accurate short read alignment with Burrows-Wheeler transform. Bioinformatics, 25(14), 1754-1760. https://doi.org/10.1093/bioinformatics/btp324HiRisePutnam, N. H., O'Connell, B., Stites, J. C., Rice, B. J., Blanchette, M., Calef, R., Troll, C. J., Fields, A. T., Hartley, P. S., Sugnet, C. W., Haussler, D., Rokhsar, D. S., & Green, R. (2016). Chromosome-scale shotgun assembly using an in vitro method for long-range linkage. Genome Research, 26(3), 342-350. https://doi.org/10.1101/gr.193474.115Minmap2Li, H. (2018). Minimap2: pairwise alignment for nucleotide sequences. Bioinformatics, 34(18), 3094-3100. https://doi.org/10.1093/bioinformatics/bty191RepeatModeler2Flynn, J. M., Hubley, R., Goubert, C., Rosen, J., Clark, A. G., Feschotte, C., & Smit, A. F. (2020). RepeatModeler2 for automated genomic discovery of transposable element families. Proceedings of the National Academy of Sciences of the United States of America, 117(17), 9451-9457. https://doi.org/10.1073/pnas.1921046117

Level of Interest

Please indicate how interesting you found the manuscript: Choose an item.

Quality of Written English

Please indicate the quality of language in the manuscript: Choose an item.

Declaration of Competing Interests

Please complete a declaration of competing interests, considering the following questions:

- Have you in the past five years received reimbursements, fees, funding, or salary from an organisation that may in any way gain or lose financially from the publication of this manuscript, either now or in the future?
- Do you hold any stocks or shares in an organisation that may in any way gain or lose financially from the publication of this manuscript, either now or in the future?
- Do you hold or are you currently applying for any patents relating to the content of the manuscript?
- Have you received reimbursements, fees, funding, or salary from an organization that holds or has applied for patents relating to the content of the manuscript?
- Do you have any other financial competing interests?
- Do you have any non-financial competing interests in relation to this paper?

If you can answer no to all of the above, write 'I declare that I have no competing interests' below. If your reply is yes to any, please give details below.

I declare that I have no competing interests

I agree to the open peer review policy of the journal. I understand that my name will be included on my report to the authors and, if the manuscript is accepted for publication, my named report including any attachments I upload will be posted on the website along with the authors' responses. I agree for my report to be made available under an Open Access Creative Commons CC-BY license (http://creativecommons.org/licenses/by/4.0/). I understand that any comments which I do not wish to be included in my named report can be included as confidential comments to the editors, which will not be published.

Choose an item.

To further support our reviewers, we have joined with Publons, where you can gain additional credit to further highlight your hard work (see: https://publons.com/journal/530/gigascience). On publication of this paper, your review will be automatically added to Publons, you can then choose whether or not to claim your Publons credit. I understand this statement.

Yes Choose an item.
